# Hemispheric asymmetry in hand preference of right-handers for passive vibrotactile perception: an fNIRS study

**DOI:** 10.1038/s41598-020-70496-y

**Published:** 2020-08-07

**Authors:** Sang Hyeon Jin, Seung Hyun Lee, Seung Tae Yang, Jinung An

**Affiliations:** 1 Division of Intelligent Robot, DGIST, 333 Techno Jungang Daero, Hyeonpung-Myeon, Dalseong-Gun, Daegu, 42988 Republic of Korea; 2 Institute of Global Health Technology, College of Health Science, Korea University, Seoul, Republic of Korea; 3 School of Mechanical Engineering, Chung-Ang University, Seoul, Republic of Korea

**Keywords:** Motor cortex, Near-infrared spectroscopy

## Abstract

Hemispheric asymmetry in hand preference for passive cutaneous perception compared to active haptic perception is not well known. A functional near-infrared spectroscopy was used to evaluate the laterality of cortical facilitation when 31 normal right-handed participants were involved in 205 Hz passive vibrotactile cutaneous stimuli on their index fingers of preferred and less-preferred hand. Passive cutaneous perception resulted that preferred (right) hand stimulation was strongly leftward lateralized, whereas less-preferred (left) hand stimulation was less lateralized. This confirms that other manual haptic exploration studies described a higher hemispheric asymmetry in right-handers. Stronger cortical facilitation was found in the right primary somatosensory cortex (S1) and right somatosensory association area (SA) during left-hand stimulation but not right-hand stimulation. This finding suggests that the asymmetric activation in the S1 and SA for less-preferred (left) hand stimulation might contribute to considerably reinforce sensorimotor network just with passive vibrotactile cutaneous stimulation.

## Introduction

Handedness is typically described as preference or performance for use of a hand^1–3^ and it is the most evident behavioral asymmetry observed in humans^2,3^. It is estimated that around 90% of the world’s population is right-handed^4^. A comprehensive analysis of cortical surface area differences between 106 left-handed subjects and 1960 right-handed subjects observed no difference in bilateral cortical surface area in left-handers compared to right-handers and found no significant evidence for associations of handedness with region-specific bilateral surface areas, or their asymmetries, for regions related to language, hand motor control, or visual processing^5^. Nevertheless, hand motor control is functionally lateralized, that is, the right hand is controlled under left sensorimotor cortex and the left hand is controlled under right sensorimotor cortex. This contralateral hand motor control was found in many studies on motor cortex activation between left- and right-handers for action execution or action imagination^6–8^.

The hand motor control of objects involves a perception and motor coupling difficult to dissociate^9^. Manual tactile perception is commonly characterized by cutaneous (passive tactile) perception and haptic (active tactile) perception. These two forms of tactile perception can be distinguished by the fact whether the motor system is involved in the manual exploration of objects. In cutaneous perception, because human skin is stimulated in stationary, only the mechanical deformation of part of the skin is coded by cutaneous mechanoreceptors. In haptic perception, the deformations of the muscles, joints, and tendons by proprioceptive receptors resulting from the exploratory movements are added to cutaneous perception.

Most studies of motor cortical facilitation for handedness have focused on the haptic perception. The fMRI studies of motor cortical asymmetry for handedness during finger tapping discovered that the more hemispheric asymmetry observed in right-handers who gained more activation in the right hemisphere than in the left hemisphere during contralateral haptic tasks^10,11^. A motor evoked potential (MEP) study of haptic perceptual asymmetry related to handedness revealed that right-handers showed greater hemispheric asymmetry in motor facilitation than left-handers and that haptic-relater enhancement in corticospinal excitability were larger when sensing with the left as compared to the right hand. In contrast, left-handers showed no evidence of clear hemispheric asymmetry in relation to the hand used in the task^12^. These results coincided with other transcranial magnetic stimulation (TMS) studies describing a greater degree of hemispheric asymmetry in right-handers, who are typically more strongly lateralized than left-handers^13,14^. Our functional near-infrared spectroscopy (fNIRS) study of cortical activation for hand preference of right-handers when performing a chopstick manipulation supported that preferred hand showed stronger cortically lateralized than less-preferred hand^15^.

In contrast, there are few studies on hemispheric asymmetry of cutaneous perception compared to those of haptic perception due to experimental difficulties regarding the quantitative and constant tactile cue display. An fMRI study of passive tactile discrimination for right-handers showed a right lateralized asymmetry but passive tactile nondiscrimination tasks mainly activated the contralateral sensorimotor cortex, regardless of the hand used^16^. An fNIRS study of sensorimotor cortex response for right-handers during manual passive touch of fingers observed that contralateral cortical activation was higher than ipsilateral one for all passive tactile stimuli on right- and left-hand^17^. The results only suggested that passive touch could activate the sensorimotor cortex, but didn’t show the hemispheric asymmetry of cutaneous perception for hand preference. From our pilot study of cortical activation observation during vibrotactile stimulation on finger, less-preferred hand showed broader cortical activation than preferred hand and it suggested that cutaneous tactile stimulation by quantitative tactile display could induce different hemispheric facilitation^18^. In this study, a solenoid resonance actuator and stimulation controller were custom-made in order to provide controllable stimulation with its constant amplitude and accurate timing. However, we needed more subjects and more detail analysis to ensure the hemispheric asymmetry of cutaneous tactile perception. Some electrical median nerve stimulation studies were reported to observe the cortical excitation for tactile perception^17,20,21^. They found that electrical stimuli to the median nerve at the wrist induced the contralateral sensorimotor cortical activation. On the one hand, optimal electrical median nerve stimulation at wrist activated somatosensory cortical and peripheral response^22^. On the other hand, natural cutaneous tactile stimulation was unlike electrical median nerve stimulation at finger from the viewpoint of whole peripheral part of the sensory afferent pathway^23^. Regardless of this controversial, if we are interested in hemispherical specialization in the cutaneous vibrotactile perception, we must direct ourselves towards the study how to naturally evoke peripheral sensory nerve action potentials mediated by Pacinian channel^24,25^.

Haptic perception accompanies cutaneous perception, that is, sensory information coded by cutaneous and proprioceptive mechanoreceptors is transmitted to the central nervous system by two separate major ascending pathways. On the aforementioned evidences of the hemispheric asymmetry of right-handers in haptic perception^10–15^ and the clue of the hemispheric asymmetry of right-handers in cutaneous perception^18^, it is postulated that preferred hand of right-handers would show stronger hemispheric asymmetry in cutaneous perception without any task-relevant conditions.

## Results

### Imaging of functional cerebral hemodynamic changes

**Figure 1:**
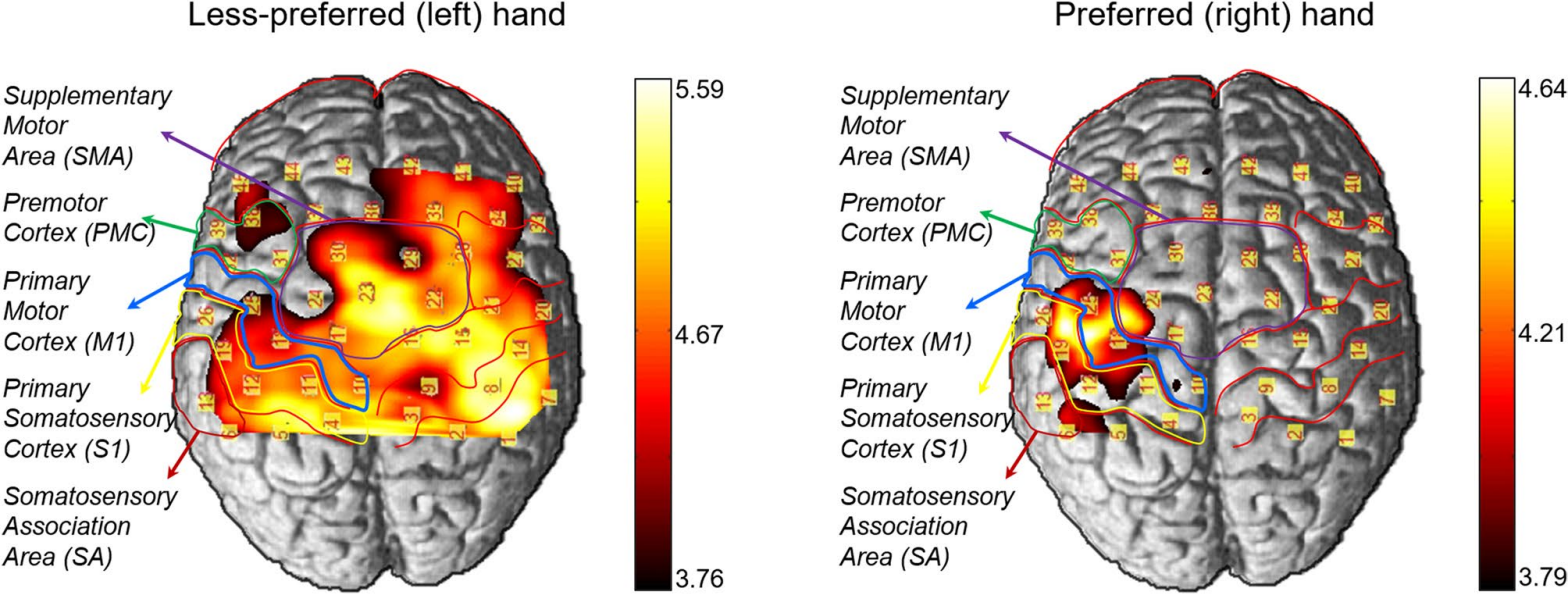
Statistical parametric mapping (SPM) of cortical activation in cutaneous perception for hand preference, EC-corrected *p* < 0.01. This figure is produced by NIRS-SPM 4.0 Copyright © 2012, Sungho Tak and Jong Chul Ye, KAIST^19^. NIRS-SPM is free software; you can distribute it and/ or modify it under the terms of the GNU General Public License (GPL) as publishing by the Free Software Foundation, either version 3 of the License, or any later version. For the copy of the GNU General Public License.

The imaging of functional cerebral hemodynamic changes was performed using the open source software package NIRS-SPM implemented in MATLAB (MathWorks, Inc., Natick, MA, USA)^19^. Figure 1 pictured the cortical activation of the oxygenated hemoglobin concentration changes (HbO) in cutaneous perception for 31 right-handers. The *p*-values correction based on expected Euler characteristics (EC) using Lipschitz–Killing curvature (LKC) were used for multiple comparisons throughout the mapping imaging analysis^26^. Significant difference in cortical activation was observed between preferred and less-preferred hand during vibrotactile stimulation. Preferred right hand showed stronger activation in the contralateral (left hemisphere) SA (CH#06, CH#13), M1 (CH#18, CH#25), and S1 (CH#12, CH#19). In contrast, Less-preferred left hand displayed broader bilateral activation than preferred right hand and especially, SMA (CH#17, CH#16, CH#23) has stronger activation.

The *t*-values of the channels in ROI were shown in Table 1. Both hands showed higher *t*-values in the contralateral side than ipsilateral side except in inferior SA. Differences in *t*-values between the contralateral and ipsilateral regions in M1 and S1 were greater for the preferred hand (1.32 ± 0.3) than for the less-preferred hand (0.71 ± 0.11). In addition, Right hand displayed higher CI values in the S1 and M1.

**Table 1:** *t*-values in the regions of interest (ROI).

		Less-preferred hand (left hand)					Preferred hand (right hand)				
		Contralateral		Ipsilateral		C/I	Contralateral		Ipsilateral		C/I
		CH	*t*-value	CH	*t*-value		CH	*t*-value	CH	*t*-value	
M1	Inferior	#21	4.67***	#25	3.86**	1.21	#25	3.95**	#21	2.19	1.80
	Middle	#15	5.09***	#18	4.27**	1.19	#18	3.96**	#15	2.61	1.52
S1	Inferior	#14	4.84***	#19	4.29**	1.13	#19	3.97**	#14	2.62	1.52
	Middle	#8	5.23***	#12	4.57***	1.14	#12	3.67*	#8	2.83	1.30
SA	Inferior	#7	4.23**	#13	3.73**	1.13	#13	2.82	#7	2.92	0.97
	Middle	#1	4.31**	#6	3.5*	1.23	#6	3.48*	#1	2.76	1.26

*M1* primary motor cortex, *S1* primary somatosensory cortex, *SA* somatosensory association area, *CH* channel number, *C/I* t-value ratio of Contralateral to Ipsilateral.

Asterisks of *, **, and *** indicated *p* < 0.05, 0.01, 0.001, respectively.

### Cerebral hemodynamic response

**Figure 2:**
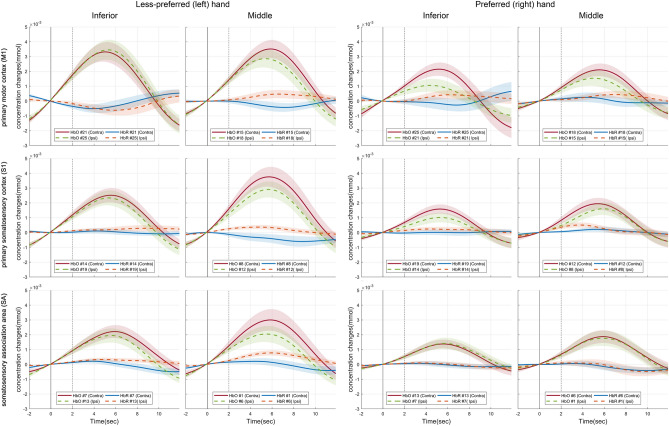
The cerebral hemodynamic time response of HbO and HbR in ROI for cutaneous perception. Shaded areas denote standard error of the mean. The window size was set to 14 s, which was from 2 s before stimulation to 12 s after stimulation. The solid and dashed line indicated the onset and end of stimulation, respectively.

Figure 2 illustrated the averaged hemodynamic changes of HbO and the deoxygenated hemoglobin concentration changes (HbR) in ROI during stimulation. Solid lines (blue and red) indicated contralateral channels, while dashed lines (green and orange) marked ipsilateral channels. In both hands, the stronger contralateral activations in the middle S1 and M1 were observed. Left hand showed higher HbO in bilateral side than right hand. Right hand displayed higher HbO in left S1 and M1. There was no distinct difference in HbR between rest and stimuli for all. Therefore, HbR was difficult to use as a feature to assess hand preference.

### Statistical significance

**Figure 3:**
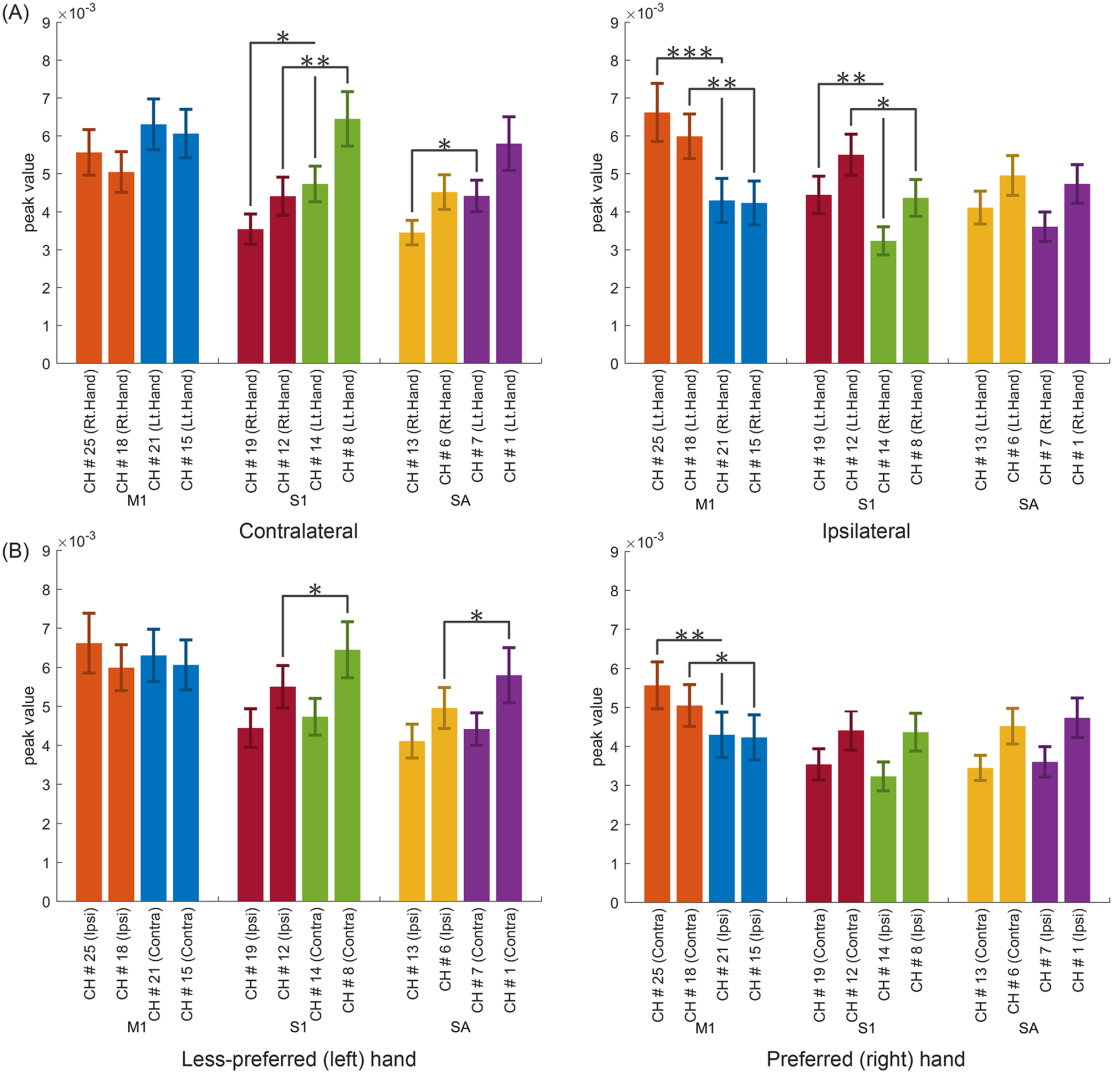
Statistical significance of difference in the peak values in HbO for right-handers in vibrotactile cutaneous perception. (**A**) comparison of hand preference according to hemispheric lateralization, (**B**) comparison of hemispheric lateralization according to hand preference. Asterisks of *, **, and *** indicated *p* < 0.05, 0.01, 0.001, respectively.

Figure 3A illustrated the statistical significance of difference in the peak values of HbO between preferred (right) and less-preferred (left) hand stimulation from the viewpoint of hemispheric lateralization. The left side of Fig. 3A showed that, in S1 and inferior SA, contralateral (right hemisphere) activations for the left-hand stimulation were significantly higher than contralateral (left hemisphere) activation for the right-hand stimulation, but no significant difference was found in contralateral activations of M1 for each hand stimulation. That is to say, right M1 activation for left-hand stimulation was not statistically different from left M1 activation for right-hand stimulation. The right side of Fig. 3A displayed that, in M1 and S1, ipsilateral (left hemisphere) activations for the left-hand stimulation went higher than ipsilateral (right hemisphere) activations for the right-hand stimulation, but no apparent difference existed for ipsilateral activation in SA. In other words, left SA activation for left-hand stimulation was not statistically different from left SA activation for right-hand stimulation.

Figure 3B represented the statistical significance of difference in the peak values of HbO between contralateral and ipsilateral hemisphere for each of preferred (right) and less-preferred (left) hand stimulation. In left-hand stimulation, no apparent lateral difference of peak values appeared in both hemispheres except in some restricted small area of S1 and SA. In right-hand stimulation, left hemispheric M1 scaled higher peak values than right hemispheric M1 but no lateral difference was shown in S1 and SA.

### Cortical lateralization

**Figure 4:**
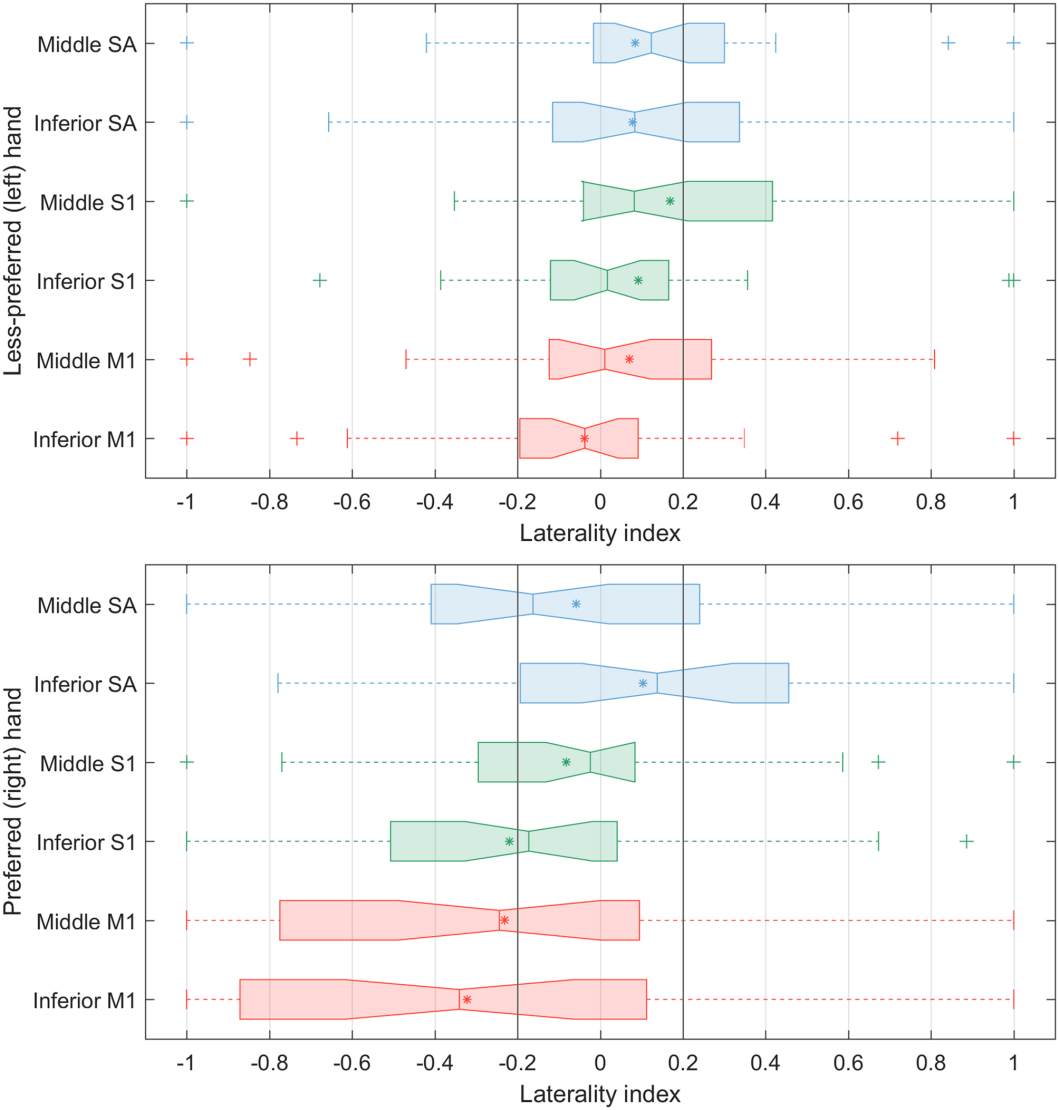
Box-and-whisker plot of the laterality index of peak values of HbO for ROI.

The laterality index (*LI*)^27^ shown in Fig. 4 was calculated from the peak values in every ROI channel for averaged data in each group. In less-preferred (left) hand stimulation, all mean and median values of *LI* with 95% confidence ranged within the threshold. The interquartile ranges (IQR) of inferior M1 and S1 were specifically in the threshold. Most of IQRs of ROI channels faced inside the threshold, except the 3^rd^ quartile (75% percentile) of middle S1 went way out of the upper bound of the threshold. On the contrary, in preferred (right) hand stimulation, all IQRs of ROI channels, which were too larger than those of less-preferred (left) hand stimulation, heavily shifted toward the left, except the inferior SA. Moreover, the mean and median values of M1 and inferior S1 were outside the lower bound of the threshold.

## Discussion

The present study examined hemispheric preference for passive vibrotactile cutaneous perception in right-handers while using either their preferred or less-preferred hand. Previous studies of motor cortical facilitation for handedness in the haptic perception (i.e., active kinesthetic perception incorporated with passive cutaneous perception) confirmed hemispheric asymmetries for hand preference^10–15^. Because generally sensory information, especially touch, is closely involved with the elaborated hand motor control, it could be expected that passive tactile sensation would contribute the haptic perception. There was also several convincing hemispheric lateralization for unimanual tactile stimulation, which showed lateralized cortical activation in the right hemisphere when the left hand was passively touched and likewise, displayed lateralized cortical activation in the left hemisphere when the right hand was passively touched^16,17^. However, they didn’t present any statistical support for the hemispheric asymmetry between two hands while tactile sensation. Our previous study of hemispheric asymmetry in tactile perception for small group of right-handed participants (*n* = 10) supplied a clue that preferred (right) hand could represent greater hemispheric asymmetry in haptic perception than less-preferred (left) hand^18^. Therefore, for larger group of subjects (*n* = 31), it was predicted that in cutaneous haptic perception, preferred hand would be more strongly lateralized than less-preferred hand and that cortical facilitation would be larger when touching with the less-preferred hand as compared to the preferred hand. In line with this prediction, the right-handers demonstrated greater cortical facilitation in sensorimotor cortex when the left hand was excited by only passive cutaneous stimulation. In conclusion, our present result suggested that if the less-preferred hand was engaged in complex manual exploration task. The evidence that manual exploration with integrating haptic information could enhance cortical facilitation in motor learning supports our claim that augmenting cutaneous tactile perception could highly contribute to reinforce motor skill learning^15,28^.

Many studies have supported that left-handers might show smaller asymmetry compared to right-handers^29–31^. Comparison of asymmetry in motor facilitation between right- and left-handers revealed that right-handers displayed greater hemispheric asymmetry than left-handers and only right-handers showed greater cortical excitation in right hemisphere when the less-preferred (left) hand was engaged in the task. In contrast, left-handers showed no evidence of clear hemispheric asymmetry in relation to the hand used in the task^12^. Based on this evidence, although the absence of left-handers in the experiment, it can be cautiously expected that left-handers could showed less cortically lateralized than right-handers in passive cutaneous tactile perception. More study involving left-handers would suggest more convincing opinion for this issue.

### Cortical activation

The results of functional cortical activation imaging visualized the hemispheric asymmetry in vibrotactile cutaneous perception for hand preference. Regardless of the hand preference, activation of the contralateral SA, S1, and M1 by passive cutaneous vibrotactile stimulation suggested that the cortical motor networks ordinarily participate in somatosensory processing^32^. In less-preferred (left) hand stimulation, bilateral SA, S1, M1, SMA, and PMC were activated. It indicated that SMA and PMC might significantly contribute to sensorimotor link in passive tactile perception for the less-preferred hand. In addition, the *t*-statistics of SPM revealed that in less-preferred hand, the contra-to-ipsilateral ratios of cortical activation in S1 and M1 were almost identical to 1. Hence, we could claim that in passive cutaneous vibrotactile perception, the less-preferred hand might be bilaterally activated, whereas the preferred hand might be more contralaterally activated.

Anatomically the middle S1 and M1 is closer to the cortical region corresponding to the digits than the inferior S1 and M1^33^. Cerebral hemodynamic time response demonstrated that the middle S1 and M1 were higher activated than the inferior S1 and M1 in both hands. It implied that the vibrotactile cutaneous perception could be discriminated corresponding to the location of vibration.

### Hemispheric asymmetry

It has been commonly accepted that each hemisphere of the primary sensorimotor cortices (S1 and M1) contains tactile or motor representation of the opposite (contralateral) side of the hand. Many anatomical and functional evidences agreed that a contralateral asymmetry within M1 was strongly lateralized by hand preference^34,35^. In agreement with contralateral asymmetry of S1 and M1, statistical significance in passive vibrotactile cutaneous perception for a group of right handers provided that less-preferred (left) hand stimulation activated bi-hemispheric S1 than preferred (right) hand stimulation, but no significant difference between both hands was found in activations of contralateral sides of M1 (i.e., right M1 activation-to-left hand stimulation vs. left M1 activation-to-right hand stimulation). These results suggested that the passive cutaneous tactile stimulation in less-preferred hand was cortically perceived higher in both hemispheric S1. Thus, it could be a reasonable opinion that passive tactile stimulation in less-preferred hand might influence on the greater facilitation of bilateral S1 than that in preferred hand.

Furthermore, direct comparison of hemispheric lateralization according to hand preference disclosed that preferred (right) hand stimulation showed distinct difference in lateralization of M1 activation, which contralateral (left hemispheric) M1 was stronger activated than ipsilateral (right hemispheric) M1. On the contrary, in less-preferred hand stimulation had no difference in lateralization of M1. It presented that preferred hand was more lateralized to the contralateral hemisphere in passive cutaneous tactile perception. In particular, it should pay attention to that cortical activation in middle S1 and SA for less-preferred hand stimulation was more significant in contralateral hemisphere than in ipsilateral hemisphere. It indicated that less-preferred hand tactile stimulation could lead to greater cortical activation in the less-proficient right hemispheric sensorimotor network.

Laterality index quantified the hemispheric asymmetry in vibrotactile cutaneous perception for hand preference. The results revealed that the cutaneous perception for less-preferred hand stimulation was bilaterally facilitated.

## Materials and methods

### Participants

We recruited 31 healthy right-handed volunteers (17 men, 14 women, age range in 19–35 years old) with no history of neurological, physical, or psychiatric illnesses. The Edinburg Handedness Inventory (EIH) was used for the evaluation of handedness^36^. The mean score on the EHI and mode were 80.93 (SD ± 16.67) and 100, respectively. Using 60 cut-off for the lateral quotient (LQ), the handedness was classified as the left-handed, the ambiguous, and the right-handed as ranged (− 100 to  − 61), (− 60 to 60), and (60 to 100), respectively. The study was approved by the Institutional Review Board (IRB) of DGIST (DGIST-170816-h-030-01), and all participants submitted the written informed consent prior to the study. The methods used in this study were performed in accordance with the guidelines approved by the mentioned IRB.

### Experimental protocol

**Figure 5:**
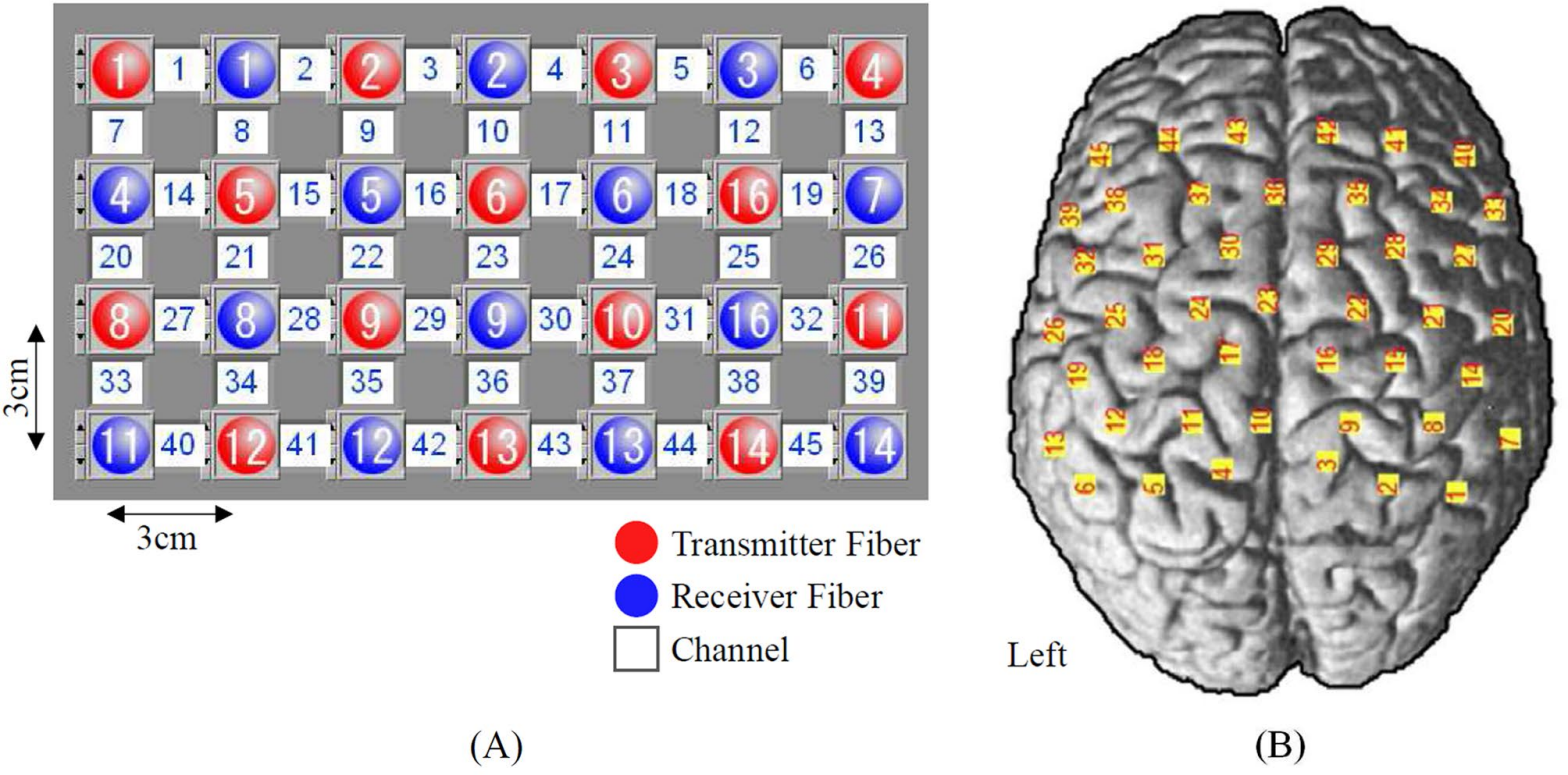
Configuration of fNIRS channel on the normal brain template. (A) 28 probes arrangement for 45 channels, (B) 45 channels placement for the cortical mapping.

We measured the cerebral hemodynamic changes for all participants to be stimulated by vibrotactile cutaneous stimulation in both hands. To present vibrations on the hands, we used a custom-made solenoid resonance actuator (SRA) with the resonant frequency of 205 Hz and the driving voltage of 5.6 V. Many different types of SRA have widely embedded in the smartphone to display crispy vibrotactile feedback^37^. Vibrotactile stimuli were presented at the distal phalanx of index finger of the preferred and less-preferred hands. The index finger was chosen as the stimulation area due to the large number of Pacinian corpuscles, which detect rapid vibration within the range of 100–500 Hz^24,25^. Participants were instructed to sit upright on a chair and not to move. Prior to the experiment, the sham vibrotactile stimulus was presented to each index finger to confirm whether subjects felt the vibration well. The experimental protocol had 2 independent sessions such as preferred and less-preferred hand stimulation. Between sessions, participants were requested for five minutes break. Each session had 10 trials. Each trial had 3 blocks consisting of rest(15 s)-task(2 s)-rest(15 s). The fNIRS system (FOIRE-3000, Shimadzu Co., Japan) was used to measure the cortical hemodynamic changes. A total of 28 probes (14 emitters, 14 receivers) were placed on the scalp through interfacing with probe holders. The probe configuration shown in Fig. 5 generated 45 channels. On the basis of 10–20 international electrode placement system, the entire channels were positioned to cover supplementary motor area (SMA) and premotor cortex (PMC), primary motor cortex (M1), primary somatosensory cortex (S1), and somatosensory association area (SA).

### Data analysis

Cerebral hemodynamic changes of HbO and HbR were gathered and processed to visualize the functional cortical imaging by statistical parametric mapping. Typically, fNIRS measurement suffered from various kinds of artifacts including motion artifacts and detrending, but no standardized artifact reduction method was established so far. Therefore, prior to SPM based cortical imaging, in order to remove artifacts, we utilized hemodynamic response function (HRF) smoothing and wavelet minimum description length (Wavelet-MDL) sequentially^15,38^. SPM based cortical imaging showed *t*-statistical mapping for relative significance between rest and task blocks in the same session. The threshold of *t*-statistical mapping in this study was set to *p* < 0.05. The functional cortical imaging and *t-*statistics were shown in Fig. 1 and Table 1, respectively.

S1, M1, and SA were identified as the ROI channels from the SPM based cortical mapping. To explore the ROI channels more closely, time responses of cerebral hemodynamic changes of HbO and HbR were performed as illustrated in Fig. 2.

The statistical significance of difference in the peak values of HbO and HbR in ROI between preferred (right) and less-preferred (left) hand should be considered to clarify the hemispherical lateralization according to the preference. Homologous cortical regions between the two cerebral hemispheres for ROI channels were shown in Table 2. Power analysis (two-tailed paired *t*-test) was performed in G*Power (version 3.1.9.6)^39^. We reported required sample size at 95% power for the study that would attempt to detect the cortical activation by statistical parametric mapping. The required sample size was calculated based on differences cortical activation for hand preference of right-handers at level of α error = 0.05 and effect size = 0.8. Thus, the required minimal total sample size would be 23 participants. Paired *t*-tests were performed after verifying normality via Kolmogorov–Smirnov test. If normality was not confirmed, non-parametric Wilcoxon signed-rank tests was used, whenever normality was not confirmed. Statistical analysis was performed using paired *t*-tests. Significant changes were accepted when *p* < 0.05.

**Table 2:** Homologous cortical regions of bihemispheric cortices for ROI channels.

	M1		S1		SA	
	Inferior	Middle	Inferior	Middle	Inferior	Middle
Channel number						
Right hemisphere	#21	#15	#14	#8	#7	#1
Left hemisphere	#25	#18	#19	#12	#13	#6

*M1* primary motor cortex, *S1* primary somatosensory cortex, *SA* somatosensory association area.

The laterality index (*LI*) was calculated to determine hemispheric preference^27^. Generally, the *LI* value is computed as the following formula:

1 $$ LI = f \times \frac{{RH_{i} - LH_{i} }}{{\left| {RH_{i} } \right| + \left| {LH_{i} } \right|}} $$

where *RH* and *LH* are the peak value of HbO at each channel *i* as measured by fNIRS for the right and left hemispheres, respectively. The factor *f* is a scaling factor that defines the range of *LI* values. Usually, *f* is held to 1 (i.e., *LI* varies between − 1 and + 1). Hemispheric contribution is typically determined by comparing *LI* to a predefined threshold (*LI*_*TH*_), according to the following rule:

2 $$ \begin{aligned} & if\,LI > LI_{TH} {:}\,right\,hemispheric\,dominance, \\ & else\,if\,LI < - LI_{TH} {:}\,left\,hemispheric\,dominance, \\ & else\,if\,\left| {LI} \right| \le LI_{TH} {:}\,bilateral\,dominance \\ \end{aligned} $$

### Ethical approval and informed consent

The study protocol was approved by the Institutional Review Board of DGIST (DGIST-170816-h-030-01), and all participants provided written informed consent.

## Supplementary information

Supplementary information.
