# Supplementary material for: Hemispheric asymmetry in hand preference of right-handers for passive vibrotactile perception: an fNIRS study
Source: Sci Rep. 2020 Aug 7;10:13423. doi: 10.1038/s41598-020-70496-y (PMC7414115; doi:10.1038/s41598-020-70496-y)
Supplement: Supplementary file 1 — Supplementary information. [file 41598_2020_70496_MOESM1_ESM.pdf]

NIRS-SPM: Statistical Parametric Mapping for Near-infrared Spectroscopy

Version 4

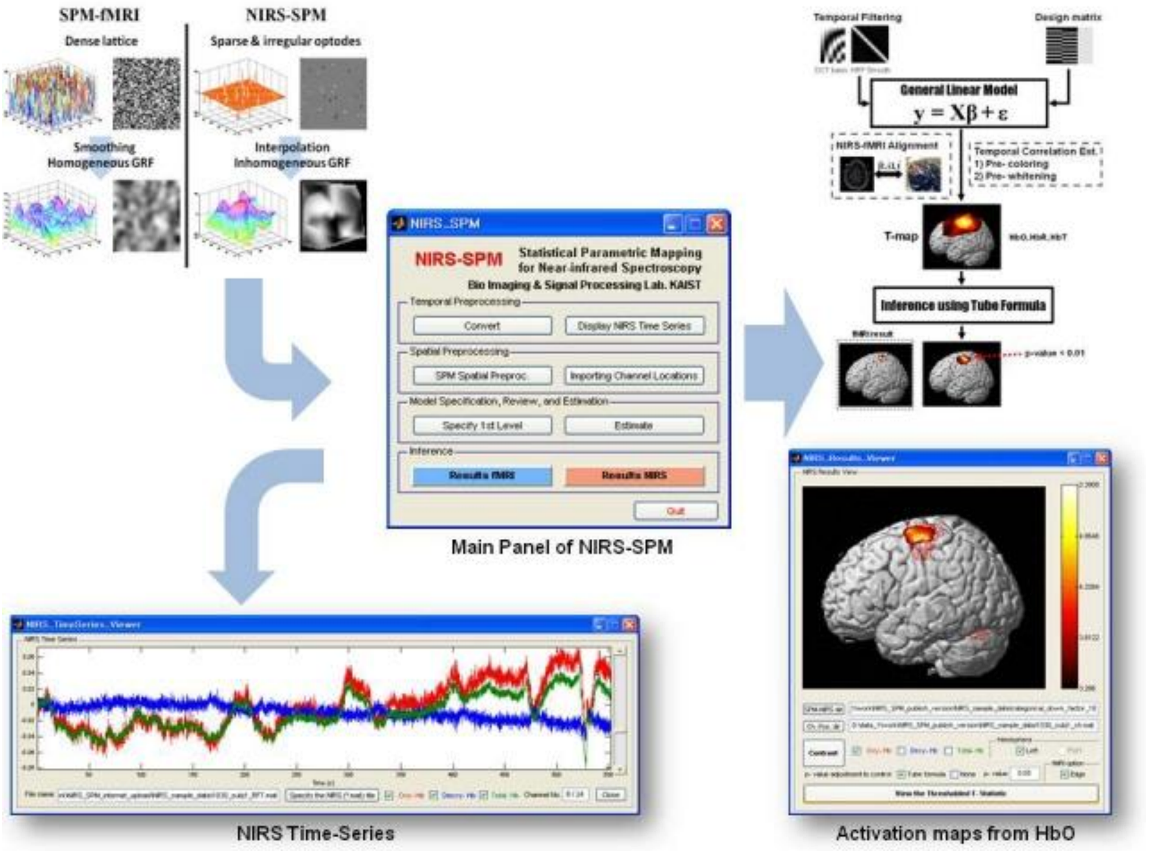

User's Guide

Release December 16th, 2011

Sungho Tak, Ph.D

Bio Imaging Signal Processing (BISP) Lab.
Dept. of Bio and Brain Engineering, KAIST
Korea

Copyright © 2011, Sungho Tak and Jong Chul Ye, KAIST

NIRS-SPM is free software; you can distribute it and/ or modify it under the terms of the GNU General Public License (GPL) as publishing by the Free Software Foundation, either version 3 of the License, or any later version. For the copy of the GNU General Public License, see <http://www.gnu.org/licenses/>

Additional Note from Authors

You can get the software freely – including source code – by downloading it here. We appreciate if you cite the following papers in producing the results using NIRS-SPM toolbox.

[1] Ye, J. C., Tak, S., Jang, K. E., Jung, J., Jang, J., 2009, NIRS-SPM: Statistical parametric mapping for near-infrared spectroscopy. NeuroImage 44, 428-447.

[2] Jang, K. E., Tak, S., Jung, J., Jang, J., Jeong, Y., and Ye, J. C., 2009, Wavelet-MDL detrending for near-infrared spectroscopy (NIRS)," Journal of Biomedical Optics, vol. 14, no. 3, pp. 1-13.

[3] Tak, S., Jang, J., Lee, K., and Ye, J. C., 2010, Quantification of CMRO2 without hypercapnia using simultaneous near-infrared spectroscopy and fMRI measurements. Physics. Med. Biol. 55, 3249-3269.

[4] Tak, S., Yoon, S. J., Jang, J. Yoo, K., Jeong, Y., and Ye, J. C., 2011, Quantitative analysis of hemodynamic and metabolic changes in subcortical vascular dementia using simultaneous near-infrared spectroscopy and fMRI measurements. NeuroImage 55, 176-184.

[5] Li, H., Tak, S., and Ye, J.C., 2012. Lipschitz Killing curvature based expected Euler characteristics for *p*-value correction in fNIRS. J. Neurosci. Meth. 204, 61-67.

NIRS-SPM is a SPM5 (http://www.fil.ion.ucl.ac.uk/spm/) and MATLAB-based software package for statistical analysis of near-infrared spectroscopy (NIRS) signals. Based on the general linear model (GLM), and Sun's tube formula / Lipschitz-Killing curvature (LKC) based expected Euler characteristics, NIRS-SPM not only provides activation maps of oxy-hemoglobin (HbO), deoxy-hemoglobin (HbR), and total-hemoglobin (HbT), but also allows for super-resolution activation localization. More details are described in Ye et al., 2009 and Li et al., 2012.

To remove the unknown global trends due to breathing, cardiac, vaso-motion, or other experimental errors, NIRS-SPM provides a wavelet-minimum description length (MDL) detrending algorithm (Jang et al., 2009).

In addition, we have developed a method to estimate cerebral metabolic rate of oxygen ($CMRO_2$) without hypercapnia by using simultaneous measurements of NIRS and fMRI (Tak et al., 2010). Using the optimization framework, many assumed parameters such as baseline hemoglobin concentration and hypercapnia can be readily estimated, which promise more accurate estimation of cerebral blood flow (CBF) and $CMRO_2$.

Some specific features of the NIRS-SPM software package are as follows:

1. **NIRS file format**: NIRS-SPM was initially developed for the analysis of optical data from the continuous wave 24-channel NIRS system (OXYMON MKⅢ, Artinis). NIRS-SPM has been recently updated for analyzing the optical density data from the other systems including the ETG 4000 (Hitachi Medical Systems), the Imagent™ (ISS, Champaign, Illinois), the NIRO 200 (Hamamatsu Photonics), DYNOT-232 (NIRx Medical Technologies, LLC.), Spectratech OEG-16, FOIRE-3000 (Shimadzu OMM), fNIR (BIOPAC Systems, Inc.), and CW6 (Techen Inc.). Furthermore, NIRS-SPM allows for the optical density changes or HbO, HbR concentration changes as for the manual input of HbO and HbR. To read other NIRS data formats from other venders, please send a data set and file format to shtak@kaist.ac.kr. We will update NIRS-SPM packages to include the data format.

2. **Spatial registration of NIRS channel locations:**
   - **NIRS-fMRI alignment:**
     NIRS channel positions in real coordinates obtained from a 3D digitizer are localized onto the cerebral cortex of an anatomical MR image using Horn's algorithm (Horn, 1987). At least three measured real coordinates of reference positions, such as a marker capsule, nasion, and inion, are necessary to elicit the relationship between the MR coordinates and real coordinates in the 3-D digitizer. Real coordinates of reference positions and optodes should be saved in a Microsoft Excel 97-2003 file format (.xls) or a text file format (.txt). Specifically, column indexes are x, y, and z coordinates, respectively. (Please refer to the sample Excel or text file.)
     NIRS-SPM provides several channel configurations such as:
     1) OXYMON MKⅢ 4x4 (1set), 2) ETG 4000 3x11 (1set), 3) ETG 4000 4x4 (2sets), 4) ETG 4000 3x5 (2sets).
   - **Stand-alone NIRS**:
     The spatial registration of NIRS channels to MNI space with MNI coordinate input is available. NIRS-SPM provides two methods for receiving MNI coordinates: 1) manual input of MNI coordinate, 2) choice of MNI coordinates from SPM template images. Also, NIRS-SPM allows the spatial registration of NIRS channels to MNI space without MRI using NFRI' fNIRS tools (Singh et al., 2005).

3. **Wavelet-MDL detrending**: Wavelet-MDL detrending algorithm effectively removes an unknown global trend due to breathing, cardiac, vaso-motion, or other experimental errors. Specifically, the wavelet transform is applied to decompose NIRS measurements into global trends, hemodynamic signals and uncorrelated noise components as distinct scales. The minimum description length (MDL) principle thereupon plays an important role in preventing over- or under- fitting and facilitates optimal model order selection for the global trend estimate. (See Jang et al., 2009)

4. **In estimating the temporal correlations**, NIRS-SPM provides both precoloring and prewhitening methods. In our data set, we showed that precoloring is more appropriate for estimating temporal correlation of NIRS data than the prewhitening method. Hence, we recommend using the precoloring method. (See Ye et al., 2009). In addition, channel-

residual covariance estimation was modified to consider channel-wise least-square residual correlation (See Li et al., 2012).

5. **In making inference about brain activation**, NIRS-SPM provides Sun's tube formula and Lipschitz-Killing curvature based expected Euler characteristics for p-value correction.. In the case of Sun's tube formula correction, *p*-values are calculated as the excursion probability of an inhomogeneous random field on a representation manifold that is dependent on the structure of the error covariance matrix and the interpolating kernels. However, Sun’s tube formula cannot be used for general random fields such as F-statistics from either individual or group analysis. To overcome these difficulties, we also provide the expected Euler characteristic approach based on Lipschitz-Killing curvature to control the family-wise error rate.
6. **$CMRO_2$ estimation without hypercapnia:** Estimation of the $CMRO_2$ and CBF is important to investigate the neurovascular coupling and physiological components in blood oxygenation level dependent (BOLD) signals quantitatively. Using an optimization framework that minimizes the differences between two-forms of relative $CMRO_2$-CBF coupling ratio from BOLD and NIRS biophysical models, unknown model parameters including hypercapnia and baseline hemoglobin concentrations are readily optimized. $CMRO_2$ and CBF relative to its baseline are then estimated accurately (See Tak et al., 2010).

7. In NIRS-SPM, a **fMRI-BOLD activation map** that has been analyzed using SPM5 can be simultaneously visualized and compared with the NIRS activation map.

■ Software Requirements

1. MATLAB (Mathworks, Natick, MA, http://www.mathworks.com). The Image Processing Toolbox is required.
   SPM5 or SPM8 (Welcome Department of Cognitive Neurology in London). It can be freely downloaded from: http://www.fil.ion.ucl.ac.uk/spm/software/.

■ Hardware Requirements

NIRS-SPM has been developed and tested on Intel® Pentium® 4 CPU 3.00 GHz, 2.00 GB RAM. However, NIRS-SPM will work on any computer with MATLAB 7 with approximately 2.0 GB RAM.
Note that the process for estimating temporal correlations requires large amount of memory. Depending on total recording time, more than 2.0 GB RAM will be required.

■ Acknowledgement

This research is supported by the IT R&D program of MKE/IITA [2008-F-021-01]. This research is also supported by the Korea Science and Engineering Foundation (KOSEF) grant funded by the Korea government (MEST) (No. 2009-0081089).

■ Updates

Although we have endeavored to develop NIRS-SPM as accurate and high quality software, there remains the possibility of some bugs. Please assist us by reporting any bugs to shtak@kaist.ac.kr. Reported bugs will be fixed in the next version of the NIRS-SPM.

■ Download and Installation Instructions

In order to download NIRS-SPM software and sample data sets, registration is required (http://bisp.kaist.ac.kr/NIRS-SPM).

After uncompressing the 'NIRS_SPM_v4.zip' file, 'NIRS_SPM_v4', 'batch_file', 'nfri_functions', 'Sample_data', and 'Documentation' directories will be created. Then, add 1) NIRS-SPM_v4 directories with sub-folders, 2) spm5 directories with sub-folders in the MATLAB path.

■ Sample Datasets

1. NIRS data files

   i. OXYMON MKⅢ sample data
      '…\Sample_data\NIRS_data_file\Artinis_OXYMON_SampleData.nir'
      Sample data were measured using OXYMON MKⅢ (24 channels, sampling frequency of 9.75 Hz). The specific behavior protocol is as follows: an initial 12s was for signal equilibrium (E). A 21-second period of finger tapping task (Finger) alternated with a 30-second period of rest (Rest); E-Rest-(Finger-Rest) x 10 repeat. The total recording time was 552 s.
   ii. Hitachi ETG 4000 sample data (1set)
      '…\Sample_data\NIRS_data_file\Hitachi_ETG4000_SampleData.csv'
   iii. Hitachi ETG 4000 sample data (2set)
      '…\Sample_data\NIRS_data_file\Hitachi_ETG4000_Set1.csv'
      '…\Sample_data\NIRS_data_file\Hitachi_ETG4000_Set2.csv'
   iv. ISS Imagent^{TM} sample data -
      '…\Sample_data\NIRS_data_file\ISS_Imagent_SampleData.log'
   v. NIRX DYNOT-232 sample data
      '…\Sample_data\NIRS_data_file\NIRX_DYNOT232_Set1.wl1' and
      '…\Sample_data\NIRS_data_file\NIRX_DYNOT232_Set2.wl2'
   vi. Spectratech OEG-16 sample data
      '…\Sample_data\NIRS_data_file\Spectratech_OEG16_SampleData.csv'
   vii. Shimadzu OMM FOIRE-3000 sample data
      '…\Sample_data\NIRS_data_file\Shimadzu_FT_Right_4x4x2.TXT'
   viii. BIOPAC fNIR sample data
      '…\Sample_data\NIRS_data_file\BIOPAC_fNIR_SampleData.oxy'
   ix. Techen CW6 sample data
      '…\Sample_data\NIRS_data_file\Techen_CW6_SampleData.nir'
      '…\Sample_data\NIRS_data_file\CW6_Hbdata_from_HomER.mat'
   x. Optical density changes sample data -
      '…\Sample_data\NIRS_data_file\OpticalDensity_SampleData.csv'
   xi. Converted HbO and HbR changes sample data
      '…\Sample_data\NIRS_data_file\HbO_HbR_SampleData.csv'

2. Spatial registration files

   i. Real coordinates of reference positions and optodes
      '…\Sample_data\Registration\RealCoordinates_xls_format.xls'
      '…\Sample_data\Registration\RealCoordinates_txt_format.txt'
      Excel\text file that contains real coordinates of 4 reference positions and 16 optode positions.

• Sample result file : '…\Sample_data\Registration\channel_NIRS_fMRI.mat'

ii. MNI coordinates of 12 optodes and corresponding 14 channels (for Stand-alone NIRS)
'…\Sample_data\Registration\MNI_standalone_optd_Singh05_NeuroImage.txt'
'…\Sample_data\Registration\MNI_standalone_ch_Singh05_NeuroImage.txt'
• Sample result file : '…\Sample_data\Registration\channel_standalone.mat'
MNI coordinates of optodes and channels was given by Singh et al., 2005.

3. Ch_config folder : Folder that contains several channel configurations.

4. T1_MRimage folder : Folder that contains anatomical (normalized) MR image.
   i. Sample anatomical image
   '…\Sample_data\T1_MRimage\uniform.img, .hdr'
   ii. Normalized anatomical image
   '…\Sample_data\T1_MRimage\wuniform.img, .hdr'
   iii. Normalization parameters
   '…\Sample_data\T1_MRimage\uniform_sn.mat'
5. fMRI_result folder: Folder that contains fMRI-SPM result files. fMRI data was simultaneously measured with NIRS data.

6. CMRO2_Est folder: Folder that contains sample dataset for estimating the $CMRO_2$ without hypercapnia from simultaneous fMRI and NIRS measurements.

▪ *How to start*

In order to run NIRS-SPM, MATLAB, spm5 (or spm 8), and NFRI' fNIRS software should be available on your system.
Please download the spm5 from http://www.fil.ion.ucl.ac.uk/spm/software/

Then, add 1) NIRS-SPM directories with sub-folders, 2) spm5 directories with sub-folders in the MATLAB path.

Start up MATLAB and type 'NIRS_SPM' at the MATLAB command window. The main panel of NIRS-SPM will then open. Analysis takes place in six stages (1) converting the optical densities to concentration changes of oxy- and deoxy- hemoglobin, (2) spatical registration of NIRS channel locations, (3) model specification, (4) detrending the unwanted global trends using wavelet-MDL algorithm or discrete cosine transform (DCT)-based high pass filtering , (5) temporal correlation estimation from precoloring or prewhitening method, (6) high resolution visualization of activated regrion from various functional contrasts (HbO, HbR, and HbT).

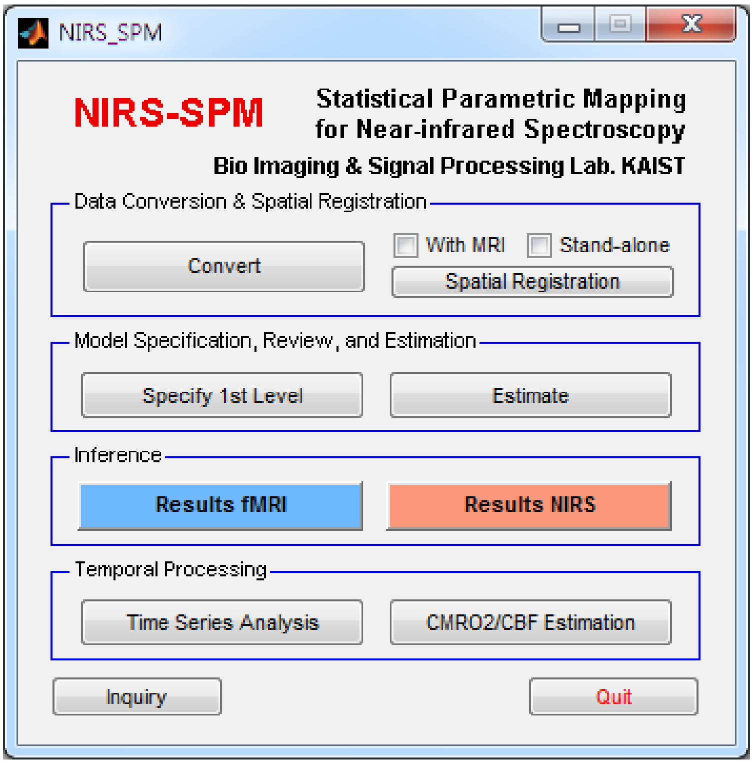

Fig. 1. Main panel of NIRS-SPM.

▪ *Convert*

In the 'Convert' routine, the modified Beer-Lambert law (Cope and Delpy, 1988) is used to calculate the concentration changes of oxy- and deoxy- hemoglobin from optical density changes. Calculated oxy- and deoxy- hemoglobin concentration changes (μM) will be saved as a '*.mat' file.
NIRS-SPM was initially developed for the analysis of optical data from the continuous wave 24-channel NIRS system (Oxymon MKIII, Artinis). NIRS-SPM has been recently updated for analyzing the optical density from the other system including the ETG 4000 (Hitachi Medical Systems), the Imagent™(ISS, Champaign, Illinois), the NIRO 200 (Hamamatsu Photonics), the DYNOT-232 (NIRx Medical Technologies, LLC.), Spectratech OEG-16, FOIRE-3000 (Shimadzu OMM) system, fNIR (BIOPAC Systems, Inc.), and CW6 (Techen Inc.). Furthermore, NIRS-SPM allows for the optical density changes or HbO, HbR concentration changes as for the manual input of HbO and HbR. To read other NIRS data formats from other venders, please send a data set and file format to shtak@kaist.ac.kr. We will update NIRS-SPM packages to include the data format.

Select the 'Convert' button in the main panel. 'NIRS_Data_Conversion' window will then open.

- OXYMON MKⅢ 24 channel system

1. Select the 'System configuration' checkbox, and choose the 'OXYMON MKⅢ' from the pop-up menu.
2. Highlight 'Sampling freq.[Hz]' and enter the corresponding sampling frequency e.g., 9.75.
3. Highlight 'Distance[cm]' which means the distance between the source and the detector and enter a specific value e.g., 3.5.
4. Highlight 'Wave length [nm]' which means the wavelength of the light sources. Then, enter a specific value e.g., 856 781.
5. Highlight 'DPF' which means the differential pathlength factor and enter a specific value e.g., 4.
6. Highlight 'correction' checkbox and choose the checkbox if you want to correct for the wavelength dependency of the differential pathlength factor (DPF). Note that NIRS-SPM allows the wavelength range of DPF correction between 704 nm and 972 nm.
7. Select 'Load' button. Use the dialog box to choose the optical density change files; e.g. …\Sample_data\NIRS_data_file\Artinis_OXYMON_SampleData.nir. Then, the conversion process starts automatically.
   NIRS-SPM supports three types of data format for OXYMON MKIII system:
   1) *.nir file (optical density changes)
      If the events exist, NIRS-SPM will start reading the dataline with the event 'A start' and finish reading the dataline with the event 'B end'.
      If the event does not exist, NIRS-SPM will read all data lines.
   2) *.xls file (hemoglobin concentration changes)
      In the 'NIRS_Data_Conversion' window, enter the sampling frequency and

select ‘Load’ button to choose the *.xls file,
e.g. …\Sample_data\NIRS_data_file\Artinis_OXYMON_Hb.xls. The selected file will then open in an Excel window. To import the oxy-Hb and deoxy-Hb concentration changes, select the worksheet in the Excel window, drag and drop the mouse over the desired range, e.g. B68:Y628, and then click OK.

3) *.txt or *.csv file (hemoglobin concentration changes)
Because the way to read *.xls file is complicated, we recommend the following process: export the Hb changes as *.txt file, or using the Excel program, save *.xls file as *.csv file.
In the ‘NIRS_Data_Conversion’ window, select the ‘Load’ button and choose the *.txt or *.csv file, ...\NIRS_data_file\Artinis_OXYMON_Hb.csv. NIRS-SPM will then read the Hb changes automatically.

8. Select ‘Save’ button. Save the concentration changes of oxy- and deoxy-hemoglobin, e.g., …\Sample_data\NIRS_data_file\Artinis_OXYMON_converted_data.mat

- Hitachi ETG-4000 System (1set)

1. Select the ‘System Configuration’ checkbox, and choose the ‘Hitachi ETG-4000 (1set)’ from the pop-up menu.
2. Select ‘Load’ button. Use the dialog box to choose the optical density change file; e.g. …\Sample_data\NIRS_data_file\Hitachi_ETG4000_SampleData.csv.
Then, conversion process starts automatically.
3. Note that ‘Sampling frequency’ and ‘Total number of channels’ will be read out in the Hitachi_ETG4000_SampleData.csv.

- Hitachi ETG-4000 System (2set)

1. Select the ‘System Configuration’ checkbox, and choose the ‘Hitachi ETG-4000 (2set)’ from the pop-up menu.
2. Select ‘Load’ button. Use the dialog box to choose the optical density file from the first set of optodes; e.g.,…\Sample_data\NIRS_data_file\Hitachi_ETG4000_Set1.csv
Then, conversion process for the first file starts automatically.
3. Use the dialog box to choose the optical density file from the second set of optodes; e.g.,..\Sample_data\NIRS_data_file\Hitachi_ETG4000_Set2.csv. Then, conversion process for the second file starts automatically.
4. Note that ‘Sampling frequency’ and ‘Total number of channels’ will be read out in the data file.

- ISS Imagent^{TM} System

1. Select the ‘System Configuration’ checkbox, and choose the ‘ISS Imagent’ from the pop-up menu.
2. Select ‘Load’ button. Use the dialog box to choose the converted HbO and HbR concentration change file;
e.g. …\Sample_data\NIRS_data_file\ISS_Imagent_SampleData.log.
Then, load data operation starts automatically. Note that ‘Sampling frequency’ will be set to be the inverse of average value of sampling interval and ‘Total number of channels’ will be read out. Sampling frequency can be manually changed.

- Hamamatsu Photonics NIRO-200 System

1. Select the ‘System Configuration’ checkbox, and chose the ‘Hamamatsu NIRO-200’

from the pop-up menu.

2. Select 'Load' button. Use the dialog box to choose the converted HbO and HbR concentration change file (e.g., '*.NI2' file).
   Then, the operation which loads the data automatically starts.

- NIRx Medical Technologies DYNOT-232 System

1. Select the 'System Configuration' checkbox, and choose the 'NIRX DYNOT-232' from the pop-up menu.
2. Highlight 'Total number of Ch.' and enter the corresponding the total number of channels e.g., 80.
3. Highlight 'Sampling freq.[Hz]' and enter the corresponding sampling frequency, e.g., 2.44.
4. Highlight 'Distance[cm]' which means the distance between the source and detector and enter a specific value e.g., 2.5.
5. Highlight 'Wave length [nm]' which means the wavelength of the light sources. Then, enter a specific value e.g., 760 830.
6. Highlight 'DPF' which means the differential pathlength factor and enter a specific value e.g., 7.15 5.98.
7. Highlight 'Extinc. Coeff.' which means the extinction coefficient and enter a specific value e.g., 1.4866 3.8437 2.2314 1.7917.
   <u>Note that the order of DPF should be the same as the order of wave length and the order of extinction coefficients should be the extinction coefficient of HbO and HbR. e.g. for 760 nm wavelength : DPF : 7.15, extinction coefficients of HbO : 1.4866 and HbR : 3.8437. for 830 nm wavelength : DPF : 5.98, extinction coefficients of HbO : 2.2314 and HbR : 1.7917</u>.
8. Select the 'Load Ch. Configuration' button and choose the '*.mat' file or '*.txt' file to contain the arrangement of channels.
   If you select the '…\Sample_data\NIRS_data_file\NIRX_DYNOT232_example.mat' file, NIRS-SPM will automatically find the channel configuration from the field 'ni.IMGlabel'.
   If you want to load the channel configuration as the text file, please see the data format from the sample file
   (e.g. …\Sample_data\Ch_config\NIRX_DYNOT232_20x32_80ch.txt' file) or see, for example, Appendix I – channel configuration.
9. Select 'Load' button and choose the '*.wl1' and '*.wl2' files, sequentially.
   e.g.,...\Sample_data\NIRS_data_file\NIRX_DYNOT232_Set1.wl1,
   …\Sample_data\NIRS_data_file\NIRX_DYNOT232_Set2.wl2.
   Then, the conversion process starts automatically.

- Spectratech OEG-16 System

1. Select the 'System Configuration' checkbox and choose the 'Spectratech OEG-16' from the pop-up menu.
2. Highlight 'Sampling freq.[Hz]' and enter the corresponding sampling frequency.
3. Select 'Load' button and choose the converted HbO and HbR concentration change file; e.g.,…\Sample_data\NIRS_data_file\OEG16Sample.csv. Then, the operation which loads the data automatically starts.

- Shimadzu OMM FOIRE-3000 System

1. Select the 'System Configuration' checkbox and choose the 'Shimadzu OMM.FOIRE-3000' from the pop-up menu.
2. Select 'Load' button and choose the converted HbO, HbR, and HbT concentration change file; e.g.,…\Sample_data\NIRS_data_file\Shimadzu_FT_Right_4x4x2.TXT. Then, the operation which loads the data automatically starts.

   For FOIRE-3000 system users, there is an instruction which introduces whole process of NIRS-SPM from data conversion of FOIRE-3000 to activation mapping. Please refer to an instruction file written by Akihiro Ishikawa, Shimadzu Corporation, Japan. e.g…\Documentation\ Instruction_FOIRE_3000_user.pdf

- BIOPAC fNIR System

1. Select the 'System Configuration' checkbox and choose the 'BIOPAC fNIR' from the pop-up menu.
2. Select 'Load' button and choose the converted HbO and HbR concentration change file; e.g. …\Sample_data\NIRS_data_file\ BIOPAC_fNIR_SampleData.oxy.
   Then, the operation which loads the data automatically starts.

- Techen Inc. CW6 System

  To calculate the hemoglobin concentration changes from CW6 data, HomER (http://www.nmr.mgh.harvard.edu/DOT/resources/homer/home.htm, Huppert et al., 2009) software is required.

1. Using HomER software, convert the optical density changes (*.nir file) to hemoglobin concentration changes and export them to *.mat file. Specifically,
   1) Raw data, e.g. …\Sample_data\NIRS_data_file\Techen_CW6_SampleData.nir, can be loaded into HomER by selecting the 'Import Data' command from the Files pull-down menu (page 27, HomER user's guide).
   2) In 'Filtering Menu', specify the cutoff frequency of low-pass filter (LPF) and high-pass filter (HPF) as 0 (Hz), and select the 'Update File' button.
   3) Click the window for probe geometry (page 5, HomER user's guide).
   4) In 'Data Display Controls', choose 'delta Concentrations' from the pop-up menu.
   5) In the figure plotting Hb concentration changes, click the right mouse button. Then, select 'Export all channels to file'. Save Hb concentration changes as *.mat file;e.g. …\Sample_data\NIRS_data_file\CW6_Hbdata_from_HomER.mat.
2. Using NIRS-SPM software,
   1) In the 'NIRS_Data_Conversion' window, select the 'System Configuration' checkbox and choose the 'HomER Software' from the pop-up menu.
   2) Select the 'Load' button. First, use the dialog box to choose *.nir file, e.g. …\Sample_data\NIRS_data_file\Techen_CW6_SampleData.nir, which provides the model parameters and channel configuration.
      Second, use the dialog box to choose *.mat file,
      e.g. …\Sample_data\NIRS_data_file\CW6_Hbdata_from_HomER.mat,
      which provides the hemoglobin concentration changes. Then, the operation loading the data automatically starts.
   3) Select 'Save' button. Save the channel configuration as *.txt file, which will be used in the step of 'Spatial registration of NIRS channels'. Then, save the hemoglobin changes as *.mat file.

- Direct input of the optical density changes

1. Select the 'Manual Input' checkbox, and choose the 'Optical density changes' from the popup menu.
2. To specify all the parameters used in modified Beer-Lambert law, NIRS-SPM provides two options;
   1) load the *.txt or *.csv file which contains the parameters. Specifically, select 'Load parameters' button. Use the dialog box to choose the parameter file; e.g., …\Sample_data\NIRS_data_file\Sample_Parameters.txt.
   2) directly enter the parameters in 'Data Conversion' window.

   Highlight 'Total number of Ch.' and enter the total number of channels, e.g., 24.
   Highlight 'Sampling freq.[Hz]' and enter the corresponding sampling frequency, e.g., 9.75.
   Highlight 'Distance[cm]' which means the distance between the source and the detector and enter a specific value, e.g., 3.5.
   Highlight 'Wave length[nm]' which means the wavelength of the light sources. Then, enter a specific value, e.g., 856 781. <u>Note that the order of wavelength should be the same as the order of wavelength saved in the optical density file (λ1, λ2).</u>
   Highlight 'DPF' which means the differential pathlength factor and enter a specific value, e.g., 4.
   Highlight 'correction' checkbox and choose the checkbox if you want to correct for the wavelength dependency of the differential pathlength factor (DPF). Note that NIRS-SPM allows the wavelength range of DPF correction between 704 nm and 972 nm.
   Highlight 'Extinc. Coeff. [$uM^{-1}mm^{-1}$] which means the extinction coefficient and enter a specific value, e.g.,
   for 856nm wavelength, extinction coefficient of HbO: 1.1885 and HbR: 0.7923,
   for 781nm wavelength, extinction coefficient of HbO: 0.7422 and HbR: 1.0803,
   the input should be 1.1885 0.7923 0.7422 1.0803.
   Note that if you don't know extinction coefficient values, please leave it blank.
   From a table of extinction coefficient depending on wavelength (Mark Cope), NIRS-SPM will then find the optimal value of extinction coefficient.

   <u>NIRS-SPM allows channel wise input of source-detector distance, DPF, wavelength, and extinction coefficient. The value of each parameter (i.e., the source-detector distance) on the specific channel can be entered into the input dialog. The order of parameter values should be the same as the order of channels. Please refer to the sample files; e.g.,…\Sample_data\NIRS_data_file\Sample_Parameters.txt.</u>

3. Select 'Load' button. Use the dialog box to choose the optical density change files (.txt or .csv); e.g. …\Sample_data\NIRS_data_file\OpticalDensity_SampleData.csv. Then, the conversion process starts automatically.

   Optical density changes (ΔOD) file format is as follows:

**ΔOD(λ1, Ch1)**	**ΔOD(λ2, Ch1)**	**ΔOD(λ1, Ch2)**	**ΔOD(λ2, Ch2)**	…
-0.0065	0.0187	0	0	…
0.0023	0.025	0.0352	-0.0245	…

-0.033	0.0187	0.0057	0.0063	⋯
0.01	0.0693	-0.0165	-0.0245	⋯
⋮	⋮	⋮	⋮	⋱

where ΔOD is optical density changes, λ1 is the first wavelength of light sources, λ2 is the second wavelength of light sources, and Ch is the number of channels.

- Direct input of the HbO and HbR concentration changes

1. Select the 'Manual Input' checkbox, and choose the 'Converted HbO and HbR changes' from the pop-up menu.
2. Highlight 'Sampling freq.[Hz]' and enter a specific value e.g., 9.75.
3. Select 'Load' button. Use the dialog box to choose the HbO and HbR concentration change files (.txt or .csv);
   e.g. ...\Sample_data\NIRS_data_file\HbO_HbR_SampleData.csv.
   Then, load data operation starts automatically.

   HbO, HbR concentration changes (ΔHbO, ΔHbR) file format is as follows:

**ΔHbO(Ch1)**	**ΔHbR(Ch1)**	**ΔHbO(Ch2)**	**ΔHbR(Ch2)**	⋯
-0.0065	0.0187	0	0	⋯
0.0023	0.025	0.0352	-0.0245	⋯
-0.033	0.0187	0.0057	0.0063	⋯
0.01	0.0693	-0.0165	-0.0245	⋯
⋮	⋮	⋮	⋮	⋱

where ΔHbO is the oxy-hemoglobin concentration changes, ΔHbR is the deoxy-hemoglobin concentration changes, and Ch is the number of channel.

Select 'Save' button. Save the concentration changes of oxy- and deoxy-hemoglobin e.g., converted_NIRS.mat.

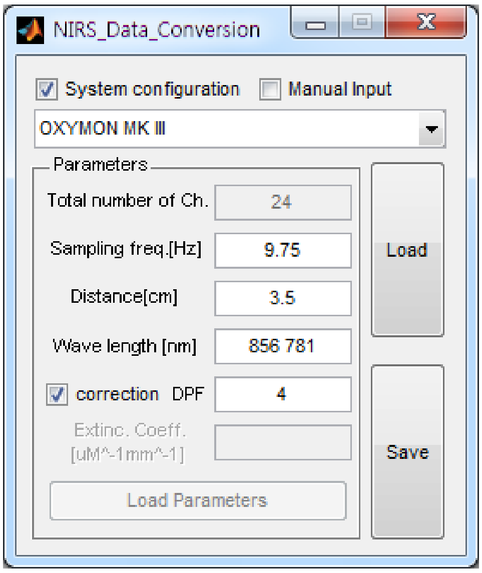

Fig. 2. The window for 'Convert' routine.

■ *Spatial Registration of NIRS Channel Locations*

NIRS-SPM allows the spatial registration of NIRS channels to MNI space with MRI (Ye et al., 2009; Horn et al., 1987) and without MRI (Singh et al., 2005) using 3D digitizer. Furthermore, the spatial registration of NIRS channels to MNI space with MRI coordinate input (for standalone) is available.

● NIRS-fMRI alignment

The relationship between the MR coordinates and the real coordinates in a 3-D digitizer is investigated from Horn's algorithm (Horn, 1987). The NIRS channel positions in real coordinates are then localized onto the cerebral cortex of an anatomical MR image. At least three measured real coordinates of reference positions, such as a marker capsule, nasion, and inion are necessary to elicit the relationship between the MR coordinates and real coordinates. Real coordinates of reference positions and optodes should be saved in a Mircosoft Excel 97-2003 file format (.xls) or a text file format (.txt). Specifically, row indexes are x, y, and z coordinates, respectively.
(Please refer to the sample excel file e.g., RealCoordinates_xls_format.xls)

1. Choose the 'With MRI' checkbox and select the 'Spatial Registration' button of the main panel. 'Indicator Locations' window will then open.
2. Setup for real coordinates:
   1) In the 'Indicator Locations' window, select the 'Load Real Coordinates' and the input window will then open. Highlight '# of reference (marker)' and enter the number of reference points (markers), e.g. 4.
   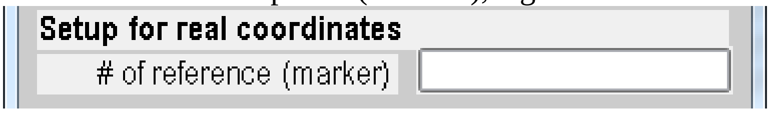

   2) Highlight '# of optodes' and enter the number of optodes, e.g., 16.
   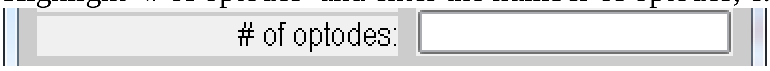

   3) Use the file selector to choose the Microsoft Excel or text file that contains real positions of references and optodes,
   e.g., …\Sample_data\Registration\RealCoordinates_xls_format.xls
   Excel file format is as follows:

	A	B	C	D	E
1	-28.3071	284.5604	388.9223	Marker Capsule 1	
2	40.7701	268.943	380.3872	Marker Capsule 2	
3	-92.8311	364.4728	342.2761	Nasion	
4	74.9538	347.1131	278.6438	Inion	
5	-36.6173	353.0598	437.3794		
6	-6.8477	336.3309	442.6411		
7	30.7733	327.3131	441.0214		
8	62.7326	326.9373	429.8878		
9	-45.9561	322.9471	421.7574		
10	-13.1585	307.2751	425.0257		
11	21.9557	297.7911	425.8241		
12	54.9315	296.3984	418.7769		
13	-49.0718	298.8532	392.3415		
14	-14.0708	285.8884	400.5728		
15	19.8753	276.8843	397.6405		
16	53.2486	278.5118	388.479		
17	-50.2078	291.424	359.6447		
18	-16.7842	276.1603	365.1786		
19	18.8958	272.6983	362.8442		
20	54.7497	277.4311	351.8033		
21	x	y	z		

The first column is x coordinates, the second column is y coordinates, and the third column is z coordinates. From the first row, the real coordinates of reference positions should be saved, and then those of optode positions should be saved in a Microsoft Excel 97-2003 file or text file format.

4) Use the file selector to choose the file containing the information of channel configuration; e.g. ...\Ch_config\Artinis_OXYMON_4x4_24ch.txt.
Appendix I describes the channel configurations in detail.

3. Load the anatomical MR images for obtaining the information of MNI coordinates of references.
   1) Select the 'MR Image' button and use the file selector to load the anatomical MR image.

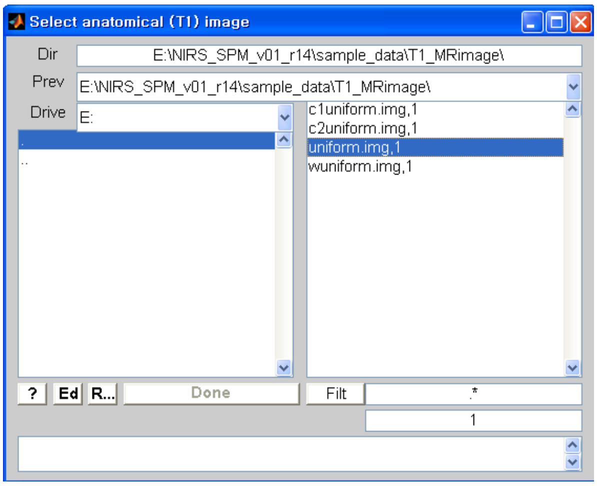

   2) Highlight 'Select anatomical (T1) image' and select the anatomical MR image file, e.g., …\T1_MRimage\uniform.img. Choose 'Done' button.
   3) Highlight 'Select normalised anatomical (wT1) image' and select the normalized anatomical MR image file e.g., …\T1_MRimage\wuniform.img. Choose 'Done'

button.
SPM5-fMRI (http://www.fil.ion.ucl.ac.uk/spm/software/spm5/) enables the anatomical T1 image to be normalized into a standard space in the 'Normalize' step. The normalized *.img scans are written to the same subdirectory as the original *.img, prefixed with a 'w' (i.e. w*.img). The parameters are saved in the '* sn.mat' file.

4) An anatomical MR image is displayed. Select reference points from the figure using the mouse and then click the + button.
Sample data has four reference points: two marker capsules, one nasion, and one inion. Specifically,
[-73.1943 36.9967 38.0649] (mm) – marker capsule #1,
[-82.059 -31.7049 36.5838] (mm) – marker capsule #2,
[-0.799051 85.0139 -15.2541] (mm) – naison,
[-8.18632 -85.632 -58.2054] (mm) – inion.

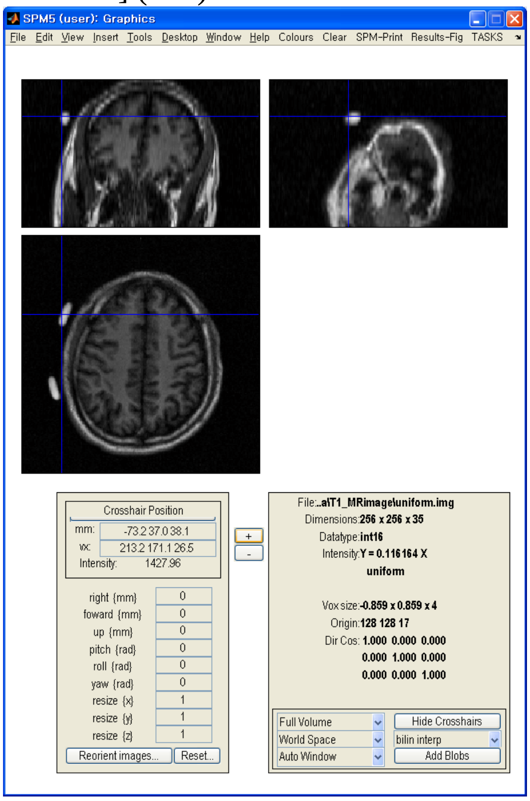

4. Select 'NIRS-MRI Alignment' button and then, choose the normalization transform parameter file e.g., …\T1_MRimage\uniform_sn.mat. Choose 'Done' button.
SPM5-fMRI (http://www.fil.ion.ucl.ac.uk/spm/software/spm5/) enables the anatomical T1 image to be normalized into a standard space in the 'Normalize'step. The parameters are saved as the '*_sn.mat' file.
5. As a result of spatial registration, the channel positions (mm) in MNI coordinates are displayed in the channel listbox.

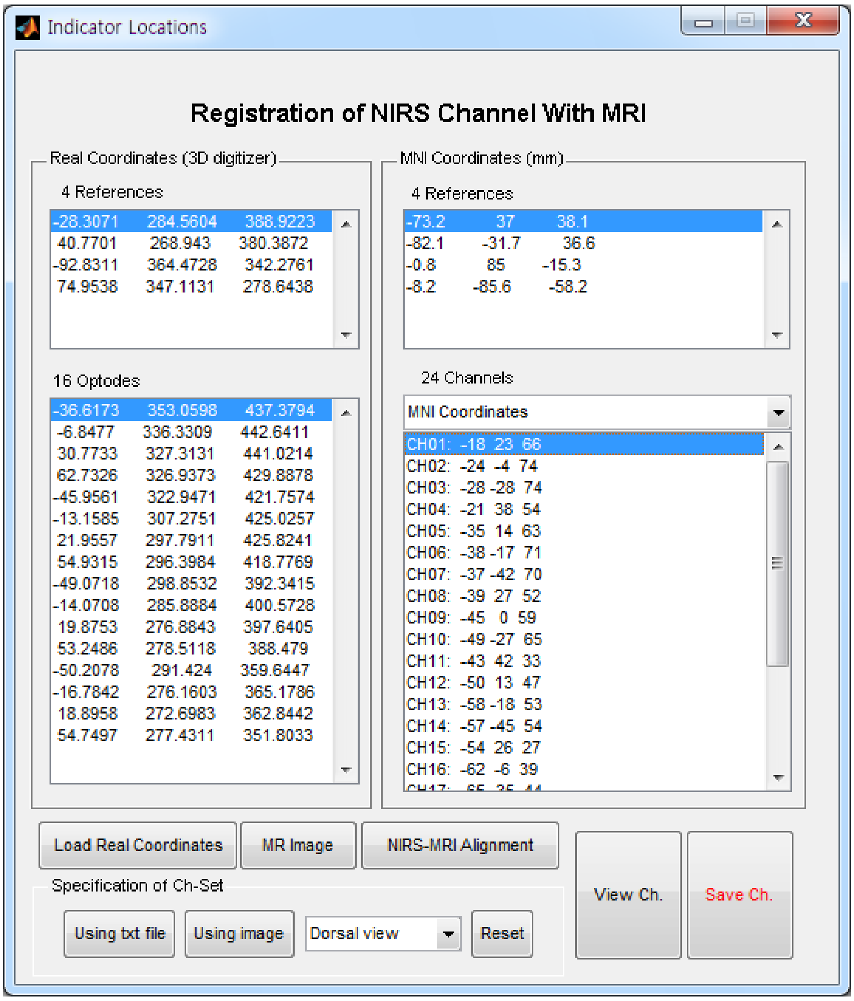

Fig. 3. ‘Indicator Locations’ window

6. Use the ‘View Ch.’ button to show the channel positions on the specific view of rendered brain.
   The sample data was recorded during the right finger tapping experiment. Since the region of interest is the left primary motor cortex, a holder cap to fix the distance between source and detector optodes was attached to the scalp around the left motor cortex. Thus, 24 channels are mainly depicted on the left lateral view of the rendered brain.

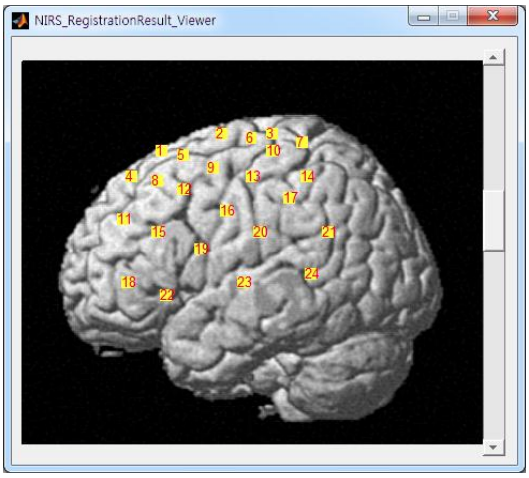

7. If the channels are divided into more than 2 sets (usually left and right hemisphere), manually specifying a region of set including specific channels is required.
   1) Use an interactive tool for selecting the channel-set within a brain image:
      i. Choose a view of the rendered brain which shows all elements (channels) within the specific set most effectively, e.g., Left lateral view.
      ii. Select the ‘Using image’ button and the brain image will then open.
      iii. Using the mouse, specify the region by selecting vertices of the polygon. When you finished positioning and sizing the polygon, create the mask by double clicking, or by right-clicking inside the region and selecting Create Mask from the context menu.

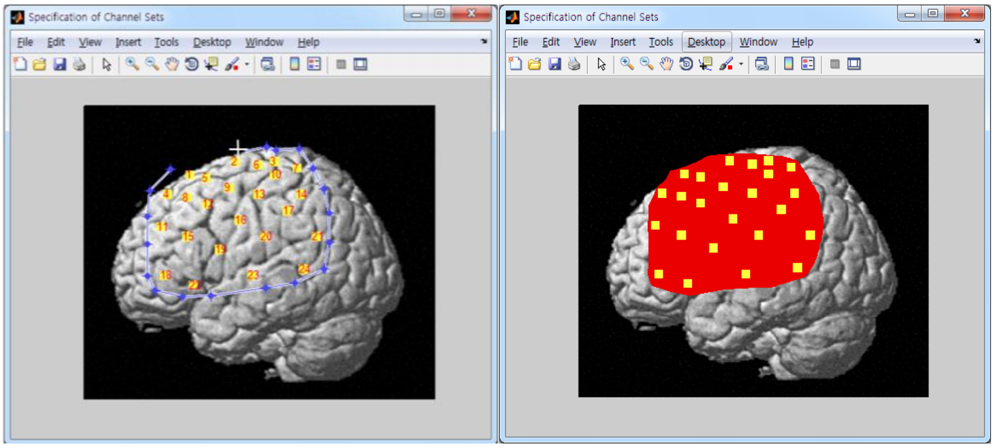

(a) (b)

(a) A polygon defined by multiple vertices, (b) A set including several channels.

      iv. Use the input dialog to specify the number of set including user-selected channels, e.g., 1. Note that in the input dialog, the information of the numbers of channels within the specified region is displayed.

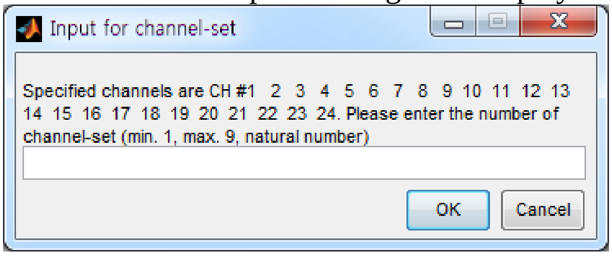

   2) Use a *.txt file which contains the information of channel-set by selecting the ‘Using txt file’ button. Refer to the sample file; e.g., …\Sample_data\Registration\Ch_Set_Artinis_OXYMON_4x4_24ch.txt.

As a result, the information of channel-set is displayed in the listbox.

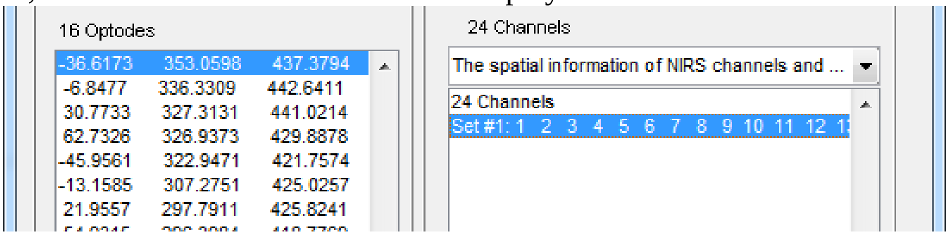

Note: If a user does not specify the channels within the set, NIRS-SPM assumes that all channels are included in one set (by default).

8. The anatomical labeling for NIRS channels is now available.
   1) Anatomic anatomical labeling (Tzourio-Mazoyer et al., 2002),
   2) Brodmann area (Chris rorden's MRIcro, Rorden et al., 2000),
   3) LPBA40 (Shattuck et al., 2007),
   4) Brodmann area (Talairach daemon, Lancaster et al., 2000).

   e.g. If you want to see the Brodmann area overlapped with the specific channel, choose the 'Brodmann Area (MRIcro)' from the pop-up menu.
   The function which was used in NIRS-SPM is 'nfri_anatomlabel.m'.

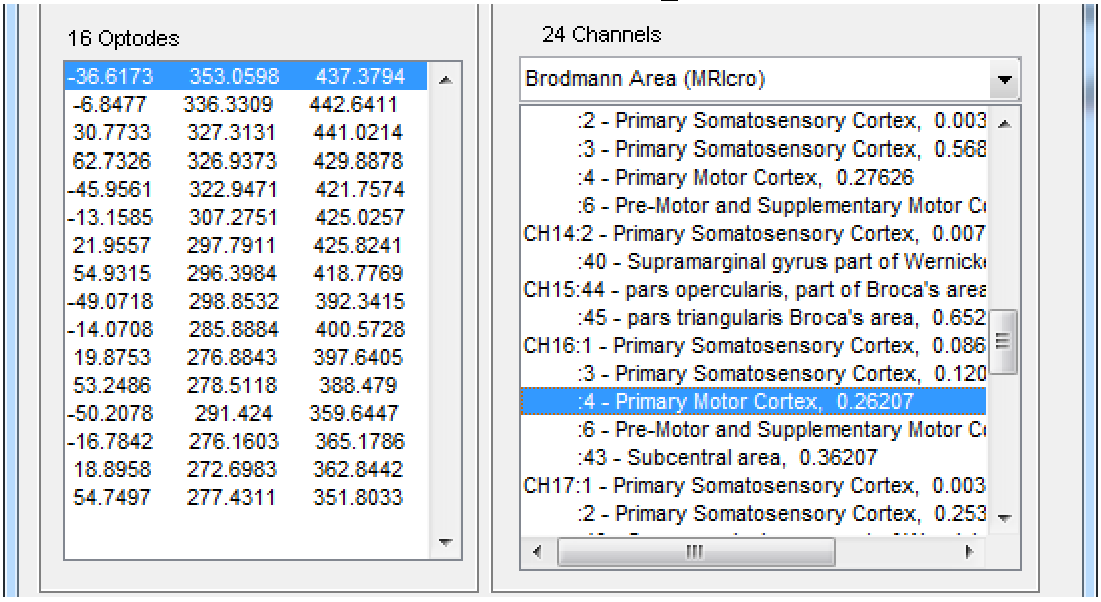

9. Select the 'Save Ch.' Button and then save the channel positions as a '*.mat' file, e.g., channel_NIRS_fMRI.mat.

● Spatial registration of stand-alone NIRS channels

NIRS-SPM provides two options for the spatial registration of stand-alone NIRS channels.
1) Spatial registration of NIRS channels to MNI coordinate using user specified MNI coordinate of optodes or channels.
2) Spatial registration of NIRS channels to MNI coordinate using 3D digitizer (NFRI' fNIRS tools, Singh et al., 2005).

■ Using user specified MNI coordinates of optodes or channels

1. Choose the 'Stand-alone' checkbox and select the 'Spatial Registration' button of the main panel. 'NIRS_Registration_Standalone' window will then open.
2. In the 'NIRS_Registration_Standalone' window, select the 'Without 3D Digitizer' checkbox.
3. Two types of inputs are available:
   (a) ***Input: MNI coordinates of optodes***
      i. Choose the 'Optodes' radio button.
      ii. Select 'Ch. Config' button. Use the dialog box to choose the channel

configuration e.g. …\Ch_config\Standalone_Singh2005_NeuroImage.txt. Channel configuration was obtained from Singh et al., 2005 (fig.2).

iii. • In the case MNI coordinates of optodes are saved as the text file, select the 'Select the file to contain MNI coordinates of NIRS optodes' button in 'NIRS_Registration_Standalone' window. Then, choose the specific file to contain MNI coordinates of optodes e.g.,…\Sample_data\Registration\MNI_standalone_optd_Singh05_NeuroImage.txt

• You can simply enter the MNI coordinates of specific optodes: In the 'NIRS_Registration Standalone' window, highlight 'MNI coordinate of specific optode' and enter the optode number and x, y, z coordinates, sequentially [Optd #, x, y, z] e.g. 1, -53, 37, 18.
Then, select the 'Add' button.

• In the case you don't know the MNI coordinates, choose the 'Select the SPM template to specify MNI coordinates' button. Then, select the specific brain template e.g., …\spm5\templates\T1.nii. The brain template will be displayed. Select the specific channel points from the figure using the mouse and then click the + button. Then, the specified coordinates will be displayed on the NIRS_Registration Standalone' window and select the 'Add' button.
MNI coordinates of optodes will appear in the listbox.

(b) ***Input: MNI coordinates of channels***

i. Choose the 'Channels' radio button

ii. • In the case MNI coordinates of channels are saved as the text file, select the 'Select the file to contain MNI coordinates of NIRS channels' button in 'NIRS_Registration_Standalone' window. Then, choose the specific file to contain MNI coordinates e.g.,…\Sample_data\Registration\MNI_standalone_ch_Singh05_NeuroImage.txt.

• You can simply enter the MNI coordinate of specific channel. In the 'NIRS_Registration Standalone' window, highlight 'MNI coordinate of specific channel' and enter the channel number and x, y, z coordinates, sequentially [Ch #, x, y, z] e.g. 1, -53, 37, 18.

• In the case you don't know the MNI coordinates, select the 'Select the SPM template to specify MNI coordinates' button in 'NIRS_Registration_Standalone' window. Then, choose the specific brain template e.g., …\spm5\templates\T1.nii. The brain template will be displayed. Select the specific channel points from the figure using the mouse and then click the + button. Then, the specified coordinates will be displayed on the NIRS_Registration Standalone' window and select the 'Add' button.
MNI coordinates of channels will appear in the listbox.

4. Select the 'Project MNI coordinate to Rendered Brain' button. Then, the window showing the channel positions on the specific view of rendered brain will open.

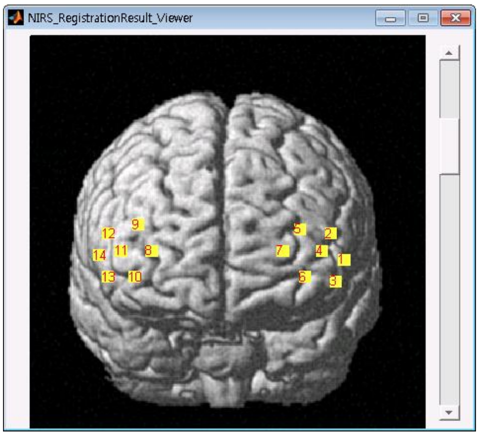

5. If the channels are divided into more than 2 sets (usually left and right hemisphere), manually specifying a region of set including specific channels is required.
   (a) Use an interactive tool for selecting the channel-set within a brain image:
      i. Choose a view of the rendered brain which shows all elements (channels) within the specific set most effectively, e.g., Frontal view
      ii. Select the 'Using image' button and the brain image will then open.
      iii. Using the mouse, specify the region by selecting vertices of the polygon. When you finished positioning and sizing the polygon, create the mask by double clicking, or by right-clicking inside the region and selecting Create Mask from the context menu.

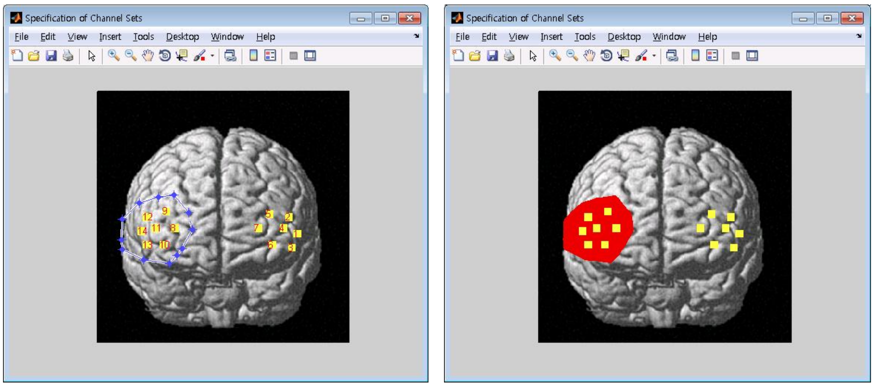

      iv. Use the input dialog to specify the number of set including user-selected channels, e.g., 1.
      Note that in the input dialog, the information of the numbers of channels within the specified region is displayed.

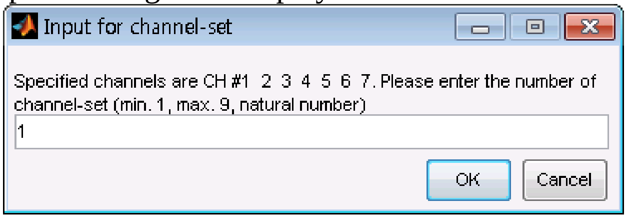

   (b) Use a *.txt file which contains the information of channel-set by selecting the 'Using txt file' button. Refer to the sample file;
   e.g. …\Sample_data\Registration\Ch_Set_Singh05_NeuroImage.txt.

As a result, the information of channel-set is displayed in the listbox.

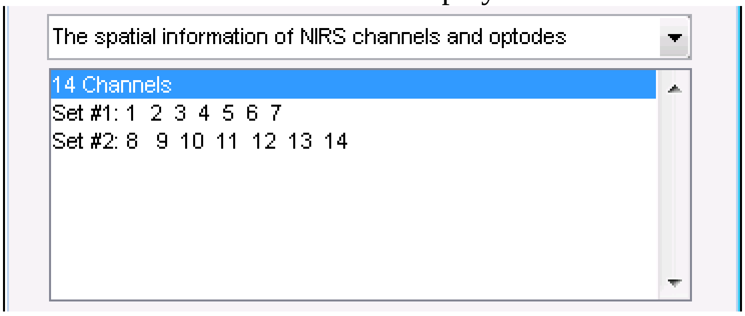

Note: If a user does not specify the channels within the set, NIRS-SPM assumes that all channels are included in one set (by default).

6. Select the ‘Save’ button and then save the channel position as a *.mat file, e.g., …\Sample_data\Registration\channel_standalone.mat.

■ **Using 3D digitizer**

In order to produce results using this function, you are also required to cite the following paper in addition to NIRS-SPM papers (Ye et al, 2009; Jang et al., 2009):

Singh, A.K., Okamoto, M., Dan, H., Jurcak, V., Dan, I., 2005, Spatial registration of multichannel multi-subject fNIRS data to MNI space without MRI. NeuroImage 27(4), 842-851.

<u>How to use the NFRI’ fNIRS tools in NIRS-SPM</u>

1. Choose the ‘Stand-alone’ checkbox and select the ‘Spatial Registration’ button of the main panel. ‘NIRS_Registration_Standalone’ window will then open.
2. In the ‘NIRS_Registration_Standalone’ window, select the ‘With 3D Digitizer’ checkbox.
3. Select the ‘Select the file (Reference position in REAL space)’ button. Use the dialog box to choose the ‘Origin’ file,
   e.g., …\Sample_data\Registration\nfri_mni_estimation_origin.csv’ file.
4. Select the ‘Select the file (Optode and Channel position in REAL space’ button. Use the dialog box to choose the ‘Others’ file,
   e.g. …\Sample_data\Registration\nfri_mni_estimation_otheres.csv’file.
   Note: The file formats of ‘Origin’ and ‘Others’ file are described at the end of this subsection.
5. Select the ‘Registration (use the NFRI function)’ button.
6. The GUI will ask you to choose the reference positions of your preference. Pick up at least four positions, and click “OK”.

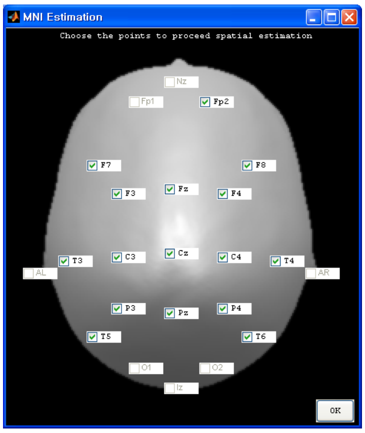

Then, the process for spatial registration starts and after a while, results are obtained as shown in Fig. 4(a)(b).

7. Select the ‘Project MNI Coordinate to Rendered Brain’ and the positions of NIRS channels on the rendered brain are then obtained as shown in Fig.4. (c).

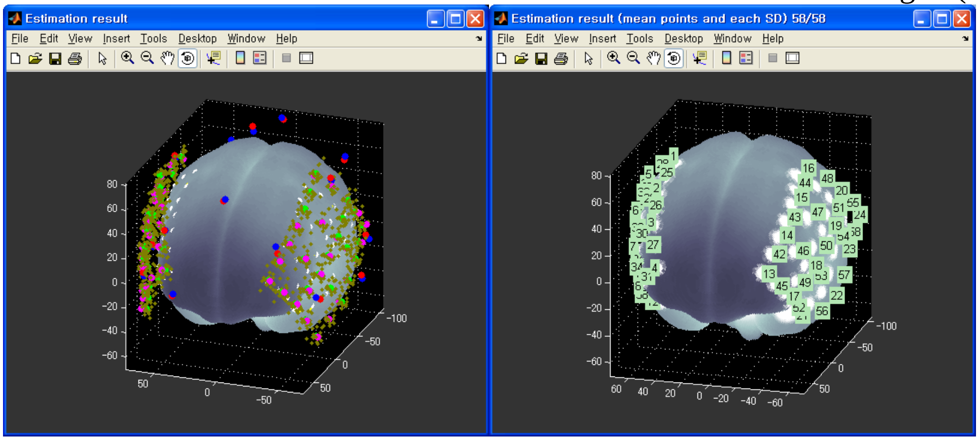

(a) (b)

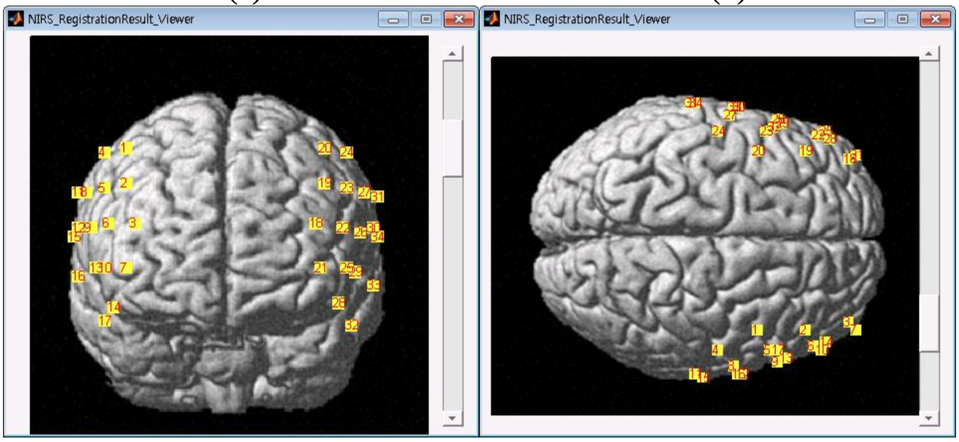

(c)

Fig. 4. The result of spatial registration of NIRS channels without MRI. (a) red dots are the real-world reference points transferred to the MNI space. Blue dots are the reference positions in the MNI space (only mean values are shown). So, if the red and blue points are located close, you can guess transformation has been successful. Brown dots indicate distribution of head surface registration among reference brains, whose mean is indicated in pink. This is projected back onto the hypothetical head surface (green), and projected on the cortical surface indicated

in white. (b) white circle regions indicate the probablistic boundary of estimation defined by standard deviation. The data numbers are indicated as appearing in the *others* file. (c) The channel that is depicted on the dorsal, frontal (ventral, occipital, lateral) views of the rendered brain

8. If the channels are divided into more than 2 sets (usually left and right hemisphere), manually specifying a region of set including specific channels is required.
   (a) Use an interactive tool for selecting the channel-set within a brain image:
      i. Choose a view of the rendered brain which shows all elements (channels) within the specific set most effectively, e.g., dorsal view.
      ii. Select the ‘Using image’ button and the brain image will then open.
      iii. Using the mouse, specify the region by selecting vertices of the polygon. When you finished positioning and sizing the polygon, create the mask by double clicking, or by right-clicking inside the region and selecting Create Mask from the context menu.
      e.g. for set #1,

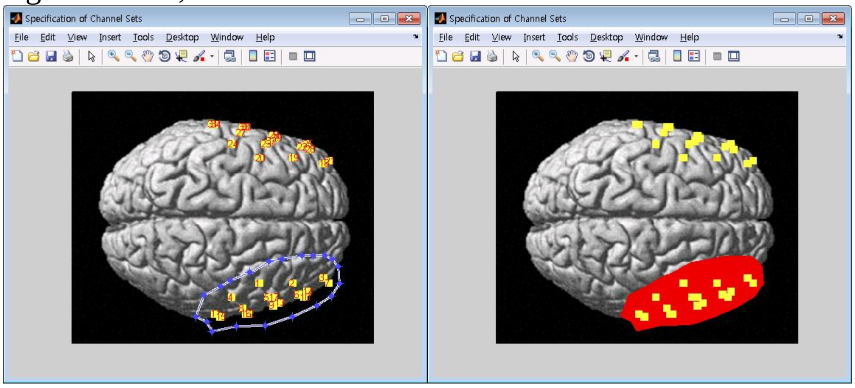

for set # 2,

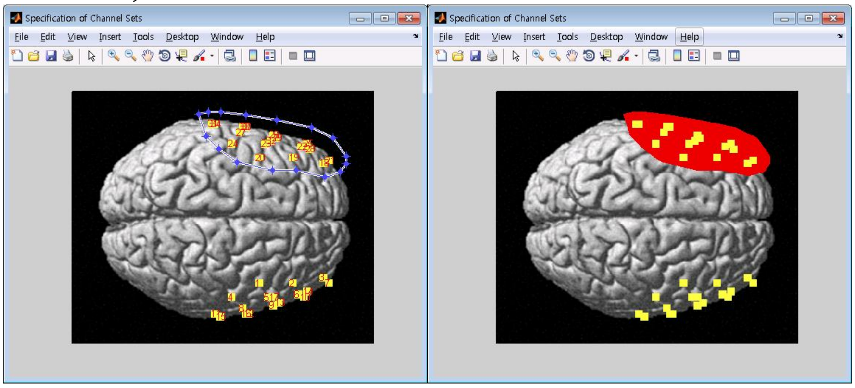

iv. Use the input dialog to specify the number of set including user-selected channels.. Note that in the input dialog, the information of the numbers of channels within the specified region is displayed.
e.g., for set #1,

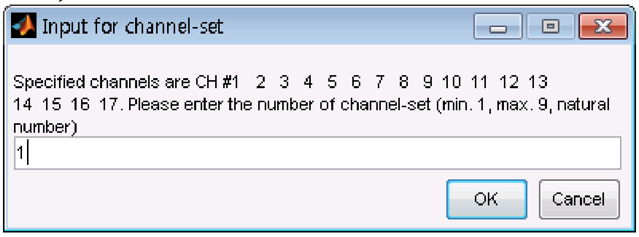

for set # 2,

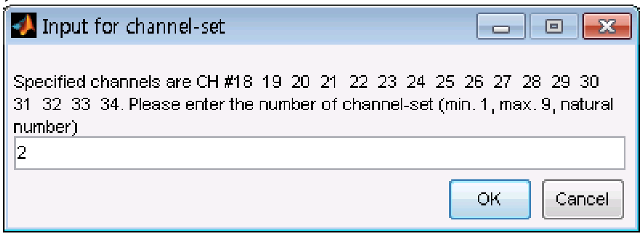

(b) Use a *.txt file which contains the information of channel-set by selecting the ‘Using txt file’ button. Refer to the sample file; e.g. …\Sample_data\Registration\Ch_Set_nfri_mni_estimation_sample.txt
As a result, the information of channel-set is displayed in the listbox.

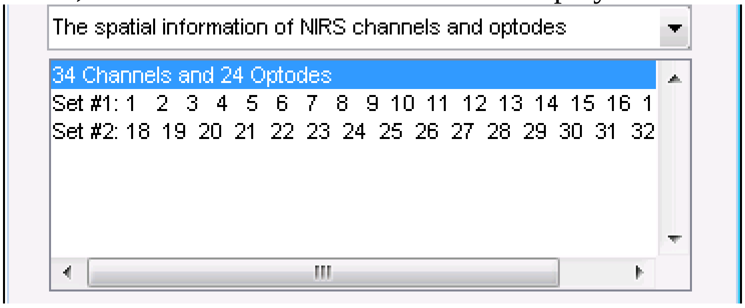

Note: If a user does not specify the channels within the set, NIRS-SPM assumes that all channels are included in one set (by default).

10. Select the ‘Save’ button and save the channel positions as a ‘*.mat’ file, e.g., …\Sample_data\Registration\channel_nfri_mni_estimation_sample.

The file format of ‘Origin’ and ‘Others’ file for real coordinates of NIRS positions

1. ‘Origin’ file for reference positions

‘The function requires at least four of the following reference positions: Iz (Inion), Nz (Nasion), AL (left preauricular point), AR (right preauricular point), Fp1, Fp2, Fz, F3, F4, F7, Cz, C3, C4, T3, T4, Pz, P3, P4, P5, T6, O1, and O2 (See Fig. 5).
Preferably, the reference positions should be spatially separated with a good balance. For example, combination of Iz, Nz, AL, AR, and Cz is great. If you measure the frontal lobe, Nz, AL, AR, Fz, and Cz might be good.
The real coordinates for the reference positions should be stored in a csv file called an ‘origin’ file. For example, in the file named “nfri_mni_estimation_origin.csv”, the first column indicates the name of the reference positions. The second, third and fourth columns indicates x, y, and z coordinates. Please insert 3D coordinates for the reference positions of your preference and leave the others blank. The function reads only the indicated reference positions.’

2. ‘Others’ file for NIRS probe positions

‘The real coordinates for the probe positions should be stored in a csv file called an ‘others’ file. For example, in the file named “nfri_mni_estimation_otheres.csv”, the first column indicates the name of the NIRS probe positions. Any name of optode positions (but not multi-bite characters) is all right. However, the name of channel positions should be as follows; CH01, CH02, CH03 etc. To estimate the channel locations, you only have to pick up an optode pair and calculate the coordinates for the midpoint, and use them as real coordinates for the channel.’

Refer to the NFRI's instruction for details.

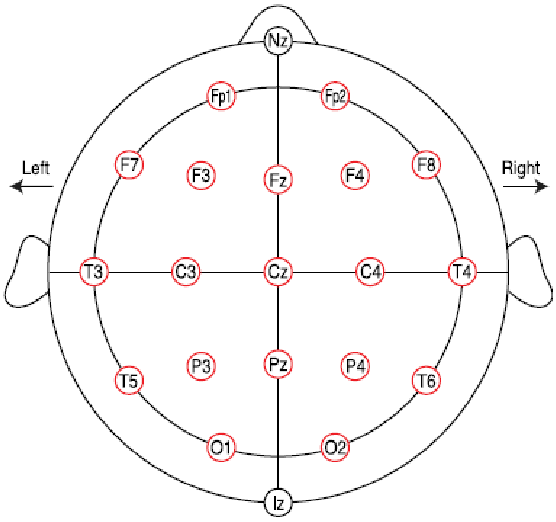

Fig. 5. Names and positions of the international 10-20 system used in this study. Nineteen standard positions in the conventional 10-20 system are shown (red circles). Nasion and inion are indicated as Nz and Iz (Okamoto et al., 2004).

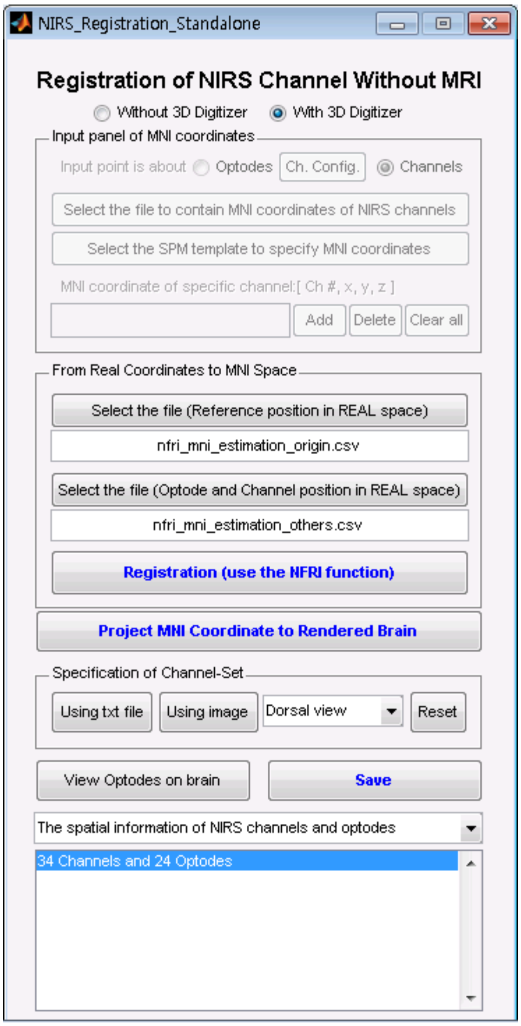

Fig. 6. The 'NIRS_Registration_Standalone' window

▪ ***Specify the 1st level***

Statistical analysis of NIRS data uses a mass-univariate approach based on the general linear model (GLM). GLM is a statistical linear model that explains data as a linear combination of an explanatory variable plus an error term. As GLM measures the temporal variational pattern of signals rather than their absolute magnitude, GLM is more robust in many cases, even for those with an incorrect diffusion pathlength factor (DPF) or with severe optical signal attenuation due to scattering or poor contact.
In 'Specify the 1st level' routine, (1) GLM design matrix, (2) hemodynamic basis function, (3) **wavelet-MDL** or DCT-based detrending method, and (4) the method of temporal correlation estimation are specified.
Several timing parameters used in constructing the design matrix are fixed as follows:
(a) Interscan interval (sec) : 1/ Sampling frequency of NIRS data (Hz)
(b) Microtime resolution : 10
(c) Microtime onset : 1

1. Select the 'Specify 1st level' button of the main panel. The 'NIRS_Specification' window will then open.
2. In the 'NIRS_Specification' window, select the 'Select nirs datafile' button and then, choose the ΔHbO, ΔHbR file,
   e.g.,…\Sample_data\NIRS_data_file\Artinis_OXYMON_converted_data.mat
   If you select the nirs file which results from the 'Time Series Analysis' routine, the parameters of 1) detrending, 2) smoothing, 3) temporal correlation correction will be fixed as the parameters used in the time series analysis.
   In this case, the method to correct for serial correlation should be the 'pre-coloring' method.
3. In the 'NIRS_Specification' window, select the 'Select SPM directory' button. Create a directory where the SPM_indiv_HbX.mat file containing the specified design matrix will be written and select it, e.g. …\Sample_data\categorical_indiv_HbO\
4. In the 'NIRS_Specification' window, select the hemoglobin checkbox, e.g., Oxy-Hb. The basis function and design matrix will be specified for the selected hemoglobin.
5. In the 'NIRS_Specification' window, select the 'Specification' button and the input window will then open.

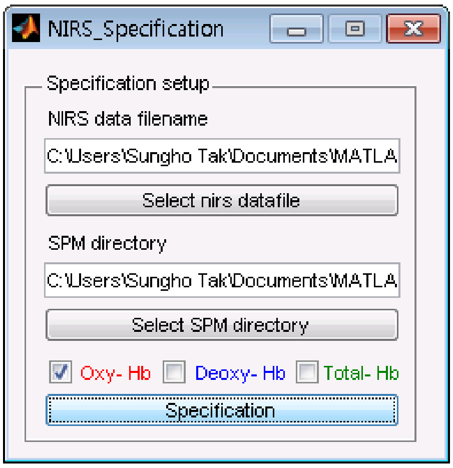

Fig. 7. 'NIRS_Specification' window

6. Highlight ‘Specify design in scans/secs’ and choose the units for design, e.g., secs. The onsets and durations of events or blocks can be specified in either scans or seconds.
7. Highlight ‘Select basis set…’ and select the basis functions to model the hemodynamic response, e.g., hrf (with time and dispersion derivatives).
8. Highlight ‘(Multiple) conditions – names, onsets, durations. Load *.mat file?’ e.g. no. If you have multiple conditions then entering the details a condition at a time is very inefficient. This option can be used to load all the required information in one-go. You will first need to create a *.mat file containing the relevant information. This *.mat file must include the following cell arrays (each 1 x n) : names, onsets, and durations. (refer to the …\NIRS_data_file\sample_multiple_condition.mat’ file)
9. Highlight ‘number of conditions/trials’ and enter the number of conditions, e.g., 1
Any number of condition (event or block) types can be specified. Block and event-related responses are modeled in exactly the same way by specifying their onsets [in terms of onset times] and their durations.
10. Highlight ‘name for condition/trial 1?’ and enter the condition name, e.g., Right finger tapping.
11. Highlight ‘vector of onsets – Right finger tapping’ and enter a vector of onset times for the experiment conditions, e.g., 42:51:501.
Note that in the case ‘the unit for design’ is ‘scans’, the vector of onsets should be (42:51:501) * sampling frequency.
12. Highlight ‘duration[s] (event=0)’ and enter the durations, e.g., 21 * ones(10,1) or 21.
Note that in the case ‘the unit for design’ is ‘scans’, the duration should be 21 * sampling frequency.

An exampling of block design sequence consists of 21 s of task (T) and 30 s of rest (R) in one cycle. The full experimental run consists of 12 s of initial signal equilibrium (E), followed by 30 s of rest, followed by ten task and rest cycle; E-R-((T-R)x10 repeat). The total recording time is 552 s.
In this case, the vector of onset times should be specified as
(unit: secs) 42:51:501 or 42 93 144 195 246 297 348 399 450 501.
(unit: scans) (42:51:501) * sampling frequency
The vector of durations should be specified as
(unit: secs) 21 * ones(10,1) or 21 21 21 21 21 21 21 21 21 21 21.
(unit: scans) 21 * ones(10,1) * sampling frequency

Block and event-related responses are modeled in the exactly the same way but by specifying their different durations.
1) If the duration is less than the interscan interval and the unit for design is scans, the duration should be specified with 0.
2) If the duration is less than 1 second and the unit for design is secs, the duration should be specified with 0.
3) If you enter a single number for the durations, it will assume that all trials conform to this duration.
4) If you have multiple different durations, then the number must match the number of onset times.

13. Highlight ‘Detrending?’ and select the detrending method

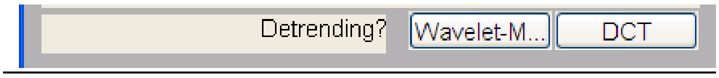

NIRS-SPM provides two options to remove an unknown global trend due to breathing, cardiac, vaso-motion, or other experimental errors.

1) Wavelet-MDL detrending algorithm
Wavelet transform is applied to decompose NIRS measurements into global trends, hemodynamic signals and uncorrelated noise components as distinct scales. The minimum description length (MDL) principle thereupon plays an important role in preventing over- or under- fitting and facilitates optimal model order selection for the global trend estimate. (See Jang et al., 2009)
If Wavelet-MDL is selected for detrending method, specify the number of trials (default = 4).

2) Discrete cosine transform (DCT) based detrending algorithm
The high-pass filter based on a DCT is currently implemented in SPM5 and the conventional detrending method.
If DCT is selected, specify the High-pass filter cut-off [seconds] (default = 128). e.g. 60.

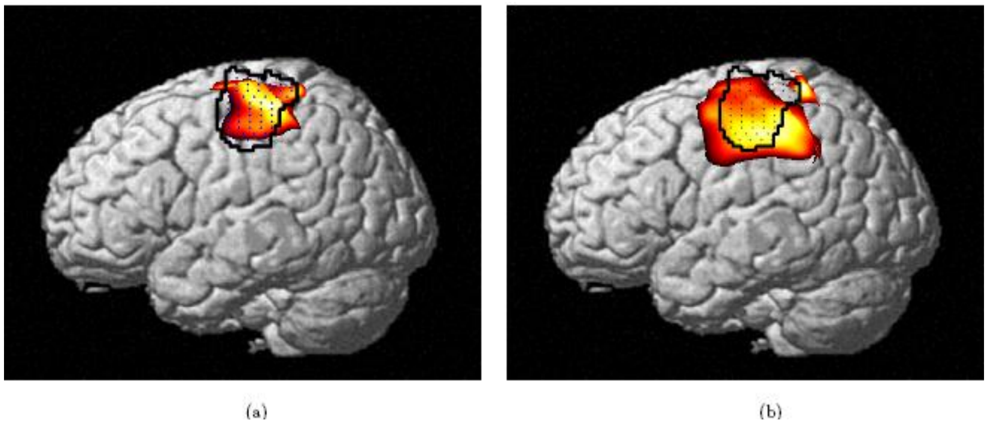

Fig. 8 NIRS-SPM group analysis result (p = 0.05) (a) Wavelet-MDL detrending, and (b) DCT-based detrending. Wavelet-MDL provides more specific activation map. Mesh-image corresponds to fMRI activation map (Jang et al, 2009).

14. Highlight ‘Low-pass filter’ and select the shape of filter.

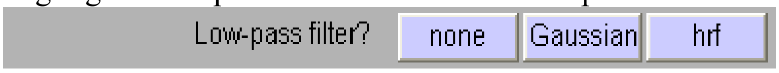

If there were no corrections for temporal autocorrelation in NIRS data, statistical inference would produce inflated results (the actual number of degrees of freedom is lower than the number of observations (scans)). NIRS-SPM provides two options to address this problem:
1. Precoloring method (Worsley and Friston et al., 1995)
In this method the intrinsic temporal correlations are swamped by an imposed

temporal correlation structure, by smoothing the data with a temporal filter that will attenuate high frequency components; hence this is a ‘low-pass filter’. The shape of this filter can be either Gaussian or hrf. Differences between these two are slight but since the transfer function of hrf is in the frequencies of modeled neuronal signals, the hrf is preferred.

2. Prewhitening method (Bullmore et al., 1996; Plichta et al., 2006, 2007; Koh et al., 2007)

This method attempts to regress out the unknown autocorrelations (AR(1) – model, see below).

If Gaussian, specify the filter width (default = 4 sec):

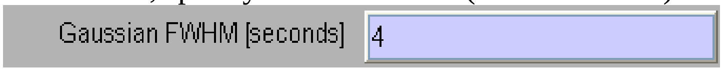

A large (wide) filter implies that attenuation will occur at lower frequencies (or, put differently, that high frequencies will be suppressed more) than with a small filter. The trade-off is that a large filter may remove interesting data while a small filter will not be adequate to impose a new (known) autocorrelation structure on the data.

Specify:

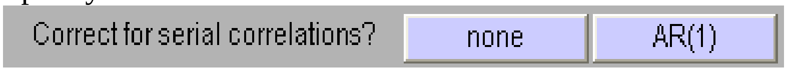

AR(1) = auto-regression (1) = modeling serial correlations by regressing out the variance explained by the previous observation (scan).

Note that

1) Either temporal smoothing (precoloring) or AR(1) (prewhitening) should be chosen, not both.
2) Precoloring method:
   Low pass filter : Gaussian or hrf → Correct for serial correlations? : none.
3) Prewhitening method:
   Low pass filter: none → Correct for serial correlations? : AR(1)
4) In our experimental data set, we showed that precoloring is more appropriate for estimating temporal correlation of NIRS data than the prewhitening method. Thus, we recommend using the precoloring method. (See Ye et al., 2009)

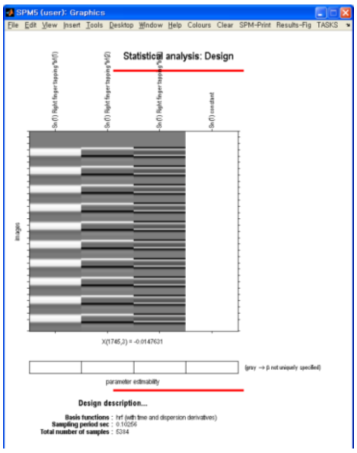

Fig. 9. Design matrix for NIRS data (HbO) from the right finger tapping task.

▪ *Estimate*

In an individual analysis, GLM parameters and temporal correlations are estimated in this routine.

● ***Individual Analysis***

1. Select the 'Estimate' button of the main panel. The 'NIRS_Estimation' window will then open.
2. In the 'NIRS_Estimation' window, select the checkbox of the analysis level, e.g., Individual Analysis.
3. In the 'NIRS_Estimation' window, select the 'Specify SPM.mat file' button and then choose the 'SPM_indiv_HbX.mat' file containing the specified design matrix, e.g., SPM_indiv_HbO.mat.
4. In the 'NIRS_Estimation' window, select the 'Estimation' button.
5. As a result of the 'Estimation' routine, the message 'Model parameter estimation has been completed' will be displayed at the MATLAB command window.

Note that

(a) In estimating the temporal correlation, the precoloring method is a memory intensive process. More than 2.00 GB RAM is required. Typically, it takes several minutes to complete the precoloring process.

If the 'out of memory' error happens, please select the following options in the 'specification' step and then perform the 'estimation' step again. Detrending? Wavelet-MDL → Low-pass filter? Gaussian or hrf → Correct for serial correlation? None.

(b) In estimating the temporal correlation, the prewhitening method is a computationally intensive process. Typically, it takes several hours to complete the prewhitening process.

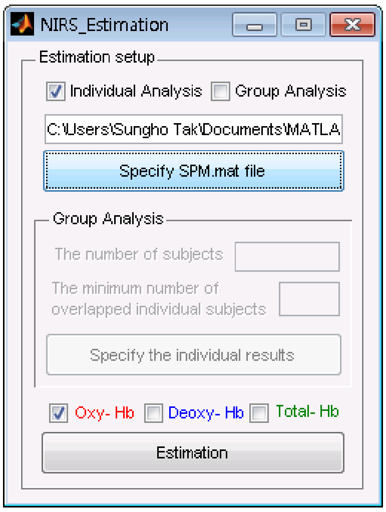

Fig. 10. 'NIRS_Estimation' window for an individual analysis

- ***Group Analysis***

1. Select the 'Estimate' button of the main panel. The 'NIRS_Estimation' window will open.
2. In the 'NIRS_Estimation' window, select the checkbox of the analysis level, e.g., Group Analysis.
3. In the 'NIRS_Estimation' window, select the 'Select SPM directory to be estimated & saved' and then, choose a directory where the 'SPM_group_(brain view e.g., dorsal)HbX.mat' file containing the design matrix and model parameters will be stored.
4. In the 'NIRS_Estimation' window, highlight the 'The number of subjects' and enter the number of subjects.
5. In the 'NIRS_Estimation' window, highlight the 'The minimum number of overlapped individual subjects' and enter the specified value.
   The interpolated individual maps are not exactly same. At the border areas of interpolated individual maps, the maximum number of the individual subjects is quite different. Thus, the region of group analysis is restricted to the areas where the number of subjects is over the user's specified value.
6. In the 'NIRS_Estimation' window, select the 'Specify the individual results' button and then, choose the file containing interpolated beta as a result of the individual session, e.g., interp_beta_(brain_view e.g., dorsal)HbX.mat. This process will be repeated as many times as the number of subjects.
   After statistical parametric mapping of an individual NIRS data has been completed, 'interp_beta_(brain_view e.g., dorsal)HbX.mat' file will be written in the directory where the 'SPM_indiv_HbX.mat' was saved (See NIRS_Results_Viewer' section).
7. In the 'NIRS_Estimation' window, select the 'Estimation' button. Then, the group analysis will automatically start.

■ *Results NIRS – Inference*

In the ‘Results NIRS’ routine, the interpolated *t*-statistic, F-statistic maps and an activation map over the threshold that is specified as above in terms of a tube formula, LKC-based expected Euler characteristics corrected *p*-value or uncorrected *p*-value are obtained.

More specifically, the NIRS signal analysis requires the excursion probability of the inhomogeneous Gaussian random field that is generated by the interpolated samples from sparsely and irregularly distributed optode measurements. Even though the excursion probability for an inhomogeneous Gaussian random field is extremely difficult to calculate in general, the excursion probability for a strikingly similar inhomogeneous random field model has been studied for the so-called global confidence region analysis of a 3D parametric shape estimation problem (Ye et al., 2006) using Sun’s tube formula (Sun, 1993). For example, the *p*- value for a one side *t*- test for oxy- or deoxy-hemoglobin concentration can be converted into the excursion probability of Gaussian random field on a two- dimensional representation manifold that is dependent on the structure of the covariance matrix and the interpolating kernels. The Gaussian SPM’s Sun’s tube formula and random field theory give the same solution (Takemura and Kuriki, 2002).

However, Sun’s tube formula cannot be used for general random fields such as F-statistics from either individual or group analysis. To overcome these difficulties, the expected Euler characteristic approach based on LKC has recently been employed for controlling the family-wise error rate in both individual and group level analysis (Li et al., 2012). Utilizing these powerful tools for calculating the excursion probability, NIRS-SPM enables inference of brain activation for HbO, HbR, and HbT with high spatial resolution (Ye et al, 2009; Li et al., 2012).

1. Select the ‘Results NIRS’ button of the main panel. The ‘NIRS Results Viewer’ window will then open.
2. In the ‘NIRS Results Viewer’ window, select the ‘SPM_nirs.mat’ button and then, choose the ‘SPM_indiv_HbX.mat’ file, e.g., SPM_indiv_HbO.mat
3. In the ‘NIRS Results Viewer’ window, select the ‘Ch. Location’ button and then, choose the ‘*.mat’ file that contains the channel positions on the SPM-brain template, e.g., channel_position.mat
   In the ‘NIRS Results Viewer’ window, highlight the ‘Views of the Rendered Brain’ and choose the specific view of the rendered brain, e.g. Left Lateral view.
4. Select the ‘Contrast’ button and ‘SPM contrast manager’ will then open. The contrast manager displays the design matrix in the right panel and lists specified contrasts in the left panel. Either ‘*t*-contrast’ or ‘*F*-contrast’ can be selected.
5. In the ‘SPM contrast manager’, select ‘Define new contrast’ to examine statistical results for condition effects.
6. In the ‘SPM contrast manager’, highlight ‘name’ and enter the contrast name e.g., Right finger tapping.
7. In the ‘SPM contrast manager’, highlight ‘type’ and select either t-contrast or F-contrast e.g., t-contrast.
8. In the ‘SPM contrast manager’, highlight ‘contrast’ and enter the contrast vector e.g., [1 0 0 0].

9. In the ‘SPM contrast manager’, press ‘submit’ and ‘OK’ button.
10. In the ‘SPM contrast manager’, select the specified contrast e.g., 001 {T} : Right finger tapping.
11. In the ‘SPM contrast manager’, Press ‘Done’.
12. After several seconds an interpolated t-map will appear in the ‘NIRS_Results_Viewer’ window.
    As a result of this routine, ‘interp_beta_leftHbX.mat’ file is written in the directory where the ‘SPM_indiv_HbX.mat’ was saved. This file will be used in the ‘Estimate-group analysis’ step. In the case the view of interest in the rendered brain is the dorsal view, ‘interp_beta_dorsalHbO.mat’ file is saved.
    Additionally, several files will be saved in the same directory:
    - interp_cov_(brain view e.g., left)HbX.mat: covariance of interpolated beta
    - interp_matrix_(brain view e.g., left).mat: interpolating matrix
    - interp_matrix_grad_(brain view e.g., left).mat: gradient of interpolating matrix
    - interp_(T or F)_(contrast #)_(brain view)_HbX.mat: interpolated t- or F-statistics
13. In the ‘NIRS_Results_Viewer’ window, highlight ‘correction’ and select the *p*-value correction method, e.g., Expected EC.
    ‘Expected EC’ is the LKC-based expected Euler characteristics, ‘Tube formula’ is Sun’s tube formula for *p*-value correction, and ‘None’ is the uncorrected *p*-value.
    Note that while the uncorrected *p*- value is too liberal, the tube formula/expected EC corrected *p*- value is reasonable for a wide range of *p*-value (Ye et al. 2009; Li et al., 2012).
14. In the ‘NIRS_Results_Viewer’ window, highlight ‘p –value’ and enter the p- value e.g., 0.05
15. In the ‘NIRS_Results_Viewer’ window, press the ‘Activation View’ button and an activation map over the threshold will finally appear.
16. In the ‘NIRS_Results_Viewer’ window, select the ‘Save Image’ button and use the file selector to save the activation map over the threshold as *.png file.

<u>In NIRS-SPM, a fMRI-BOLD activation map that has been analyzed using SPM5 can be simultaneously visualized and compared with the NIRS activation map.</u>

■ *Results fMRI*

In the ‘fMRI Results Viewer’ routine, a fMRI-BOLD activation map that has been analyzed using SPM5 is visualized.

1. Select the ‘Results fMRI’ button of the main panel.
2. Select the SPM.mat file that was created from SPM5,
   e.g., …\Sample_data\fMRI_result\SPM.mat.
3. In the ‘SPM contrast manager’, select ‘Define new contrast’ to examine statistical results for condition effects.
4. Highlight ‘name’ and enter the contrast name e.g., Right finger tapping
5. Highlight ‘contrast’ and enter the contrast vector e.g., [1 0 0 0].
6. Press ‘submit’ and ‘OK’ button.
7. Select the specified contrast e.g., 001 {T} : Right finger tapping and press ‘Done’.
8. In the input window, highlight ‘mask with other contrast(s)’ and specify ‘no’.
9. In the input window, highlight ‘title for comparison’ and enter the tile of condition e.g., ‘Right finger tapping’

10. In the input window, highlight ‘p value adjustment to control’ and specify it e.g., ‘FWE’.
11. In the input window, highlight ‘p value (family-wise error)’ and enter the p- value e.g., 0.05
12. In the input window, highlight ‘& extent threshold {voxels}’ and enter ‘0’.
13. In the input window, highlight ‘Style’ and specify ‘old’. An BOLD activation map over the threshold will finally appear.

In order to compare the NIRS activation region with the BOLD activation region more intensively, NIRS-SPM finds edges of BOLD activation and then adds them to the NIRS activation map. Note that both NIRS activation and BOLD activation should be simultaneously displayed on each viewer.

1. In the ‘NIRS Results Viewer’ window, select the checkbox e.g., ‘Overlaid with fMRI activation’.
2. In the ‘NIRS Results Viewer’ window, press ‘Activation view’.

From the ‘Specify 1st level’ routine to ‘Results NIRS’, precisely the same approach can be applied for deoxy- and total- hemoglobin.

Figures

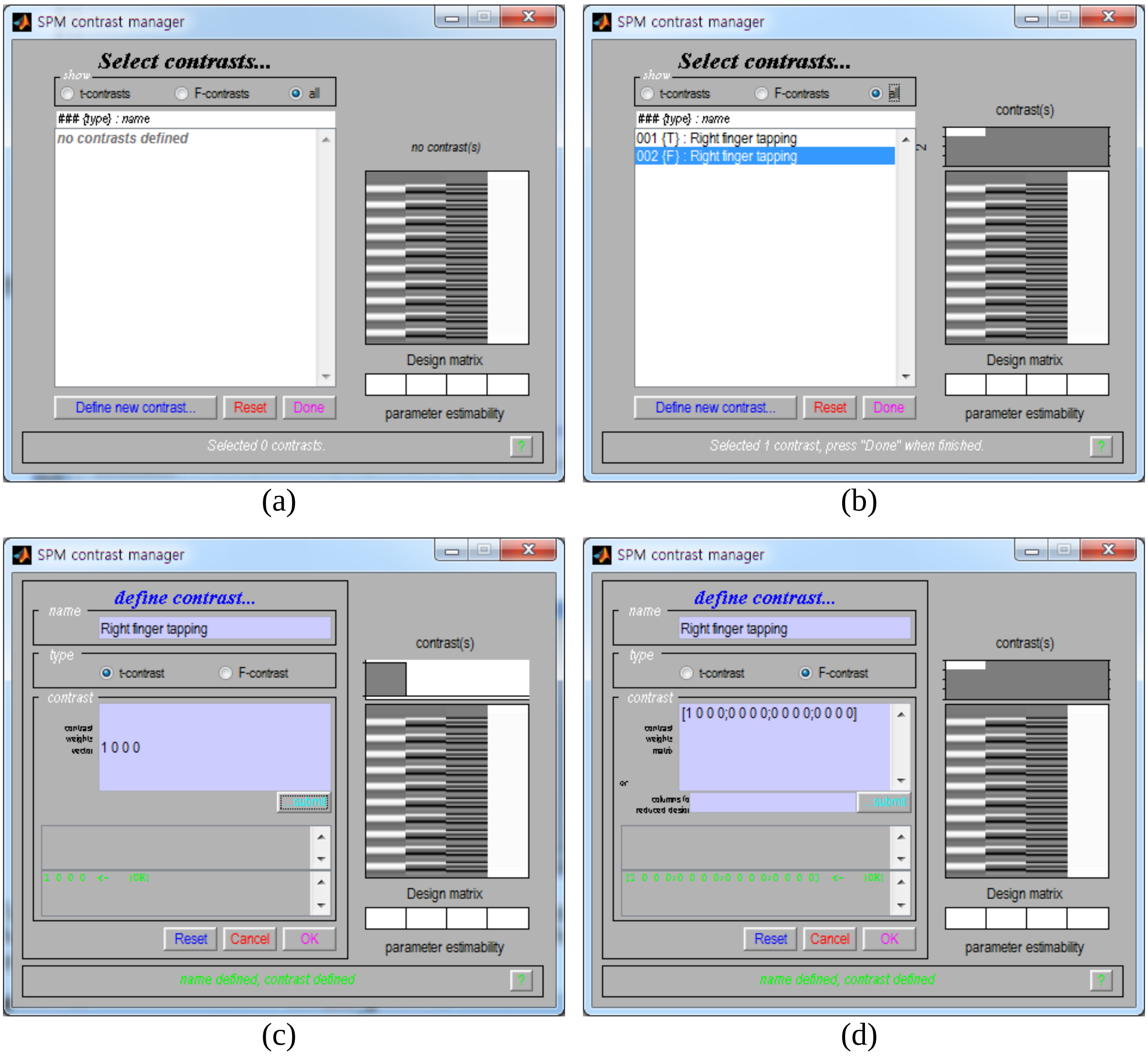

Fig. 11 Contrast manager. A contrast is entered by specifying the numeric values in the lower window and the name in the upper window. After contrasts have been specified, they can be selected.

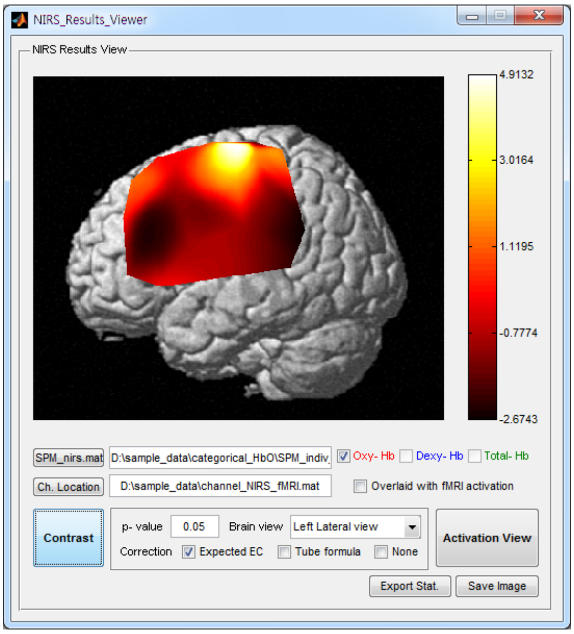

(a)

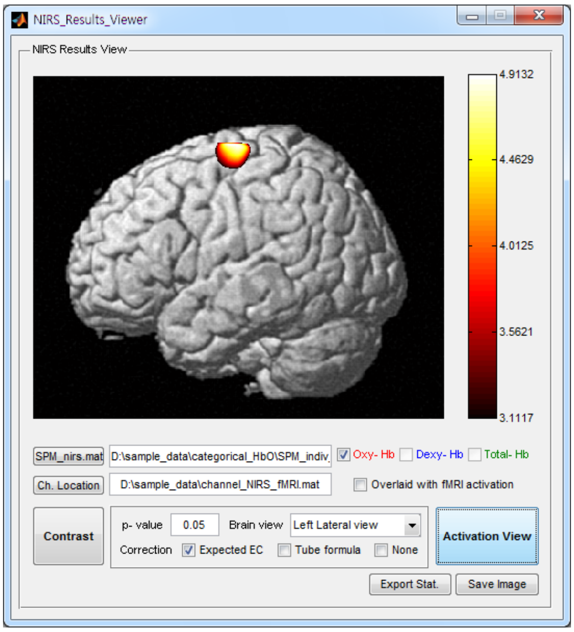

(b)

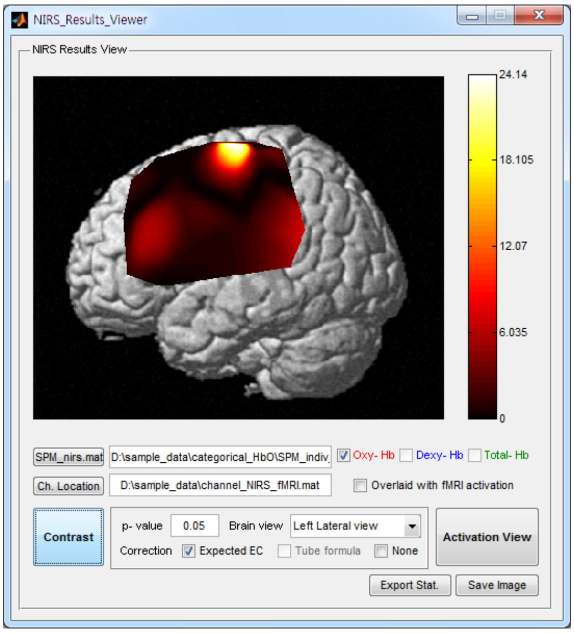

(c)

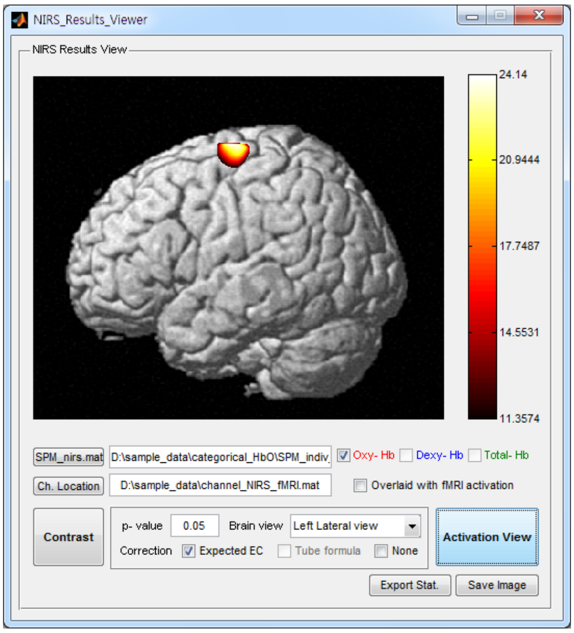

(d)

Fig. 12. ‘NIRS Results Viewer’ window. (a) Interpolated *t*-statistic map and (b) activation map of the lateral view found by HbO ($p < 0.05$, LKC-based expected EC correction). (c) Interpolated F-statistic map and (d) activation map of the lateral view found by HbO ($p < 0.05$, LKC-based expected EC correction).

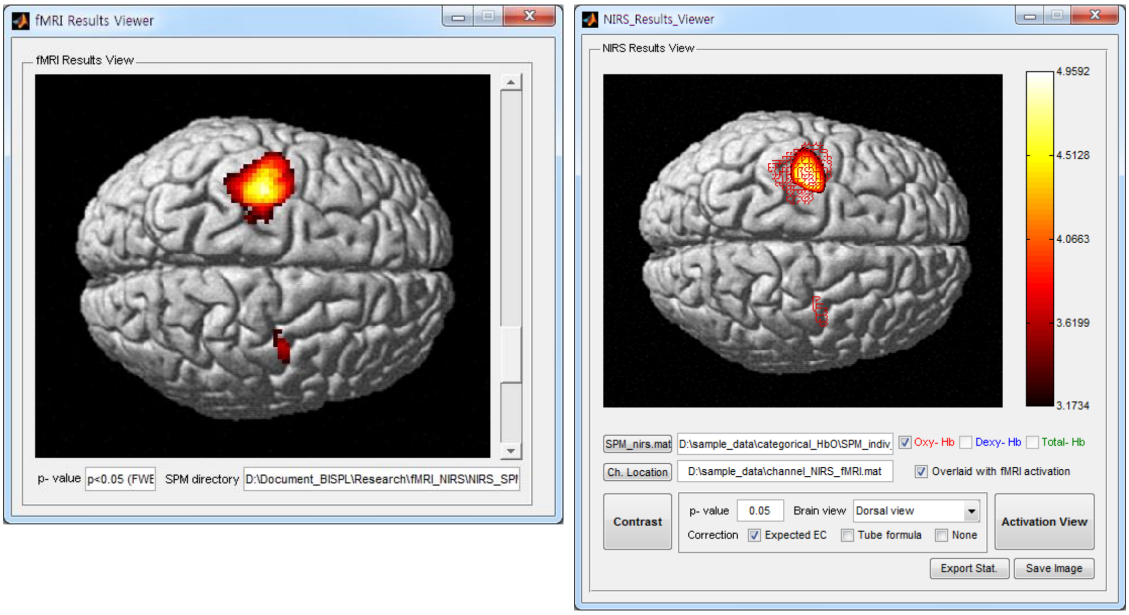

Fig. 13. 'fMRI Results Viewer' window and 'NIRS Results viewer' window. Left: Activation map of BOLD ($p<0.05$, random field correction), Right: activation map of HbO ($p<0.05$, LKC-based expected EC correction), red line: edge of BOLD activation.

▪ *Time Series Analysis*

In the 'Time Series Analysis' routine, several temporal processing of NIRS and fMRI signals are available;
1) Temporal smoothing using canonical hemodynamic response function (hrf) or Gaussian kernel (Friston et al., 2000, 2006),
2) Removing the global bias using Wavelet-MDL detrending algorithm (Jang et al., 2009) or discrete cosine transform (DCT) based detrending algorithm (Friston et al., 2006),
3) ROI analysis which plots the average time series among the certain channels or certain time-range,
4) Overlaying the predictor model and changing the limits of x- and y-axis for visual comparison,
5) Baseline correction as the initial time point or time-range.
After processing, the signals can be plotted in the 'NIRS_TimeSeries_Viewer' window and saved as '*.mat' file.
Note: fMRI time-series processed in this routine will be used in the 'CMRO2/CBF Estimation without Hypercapnia'.

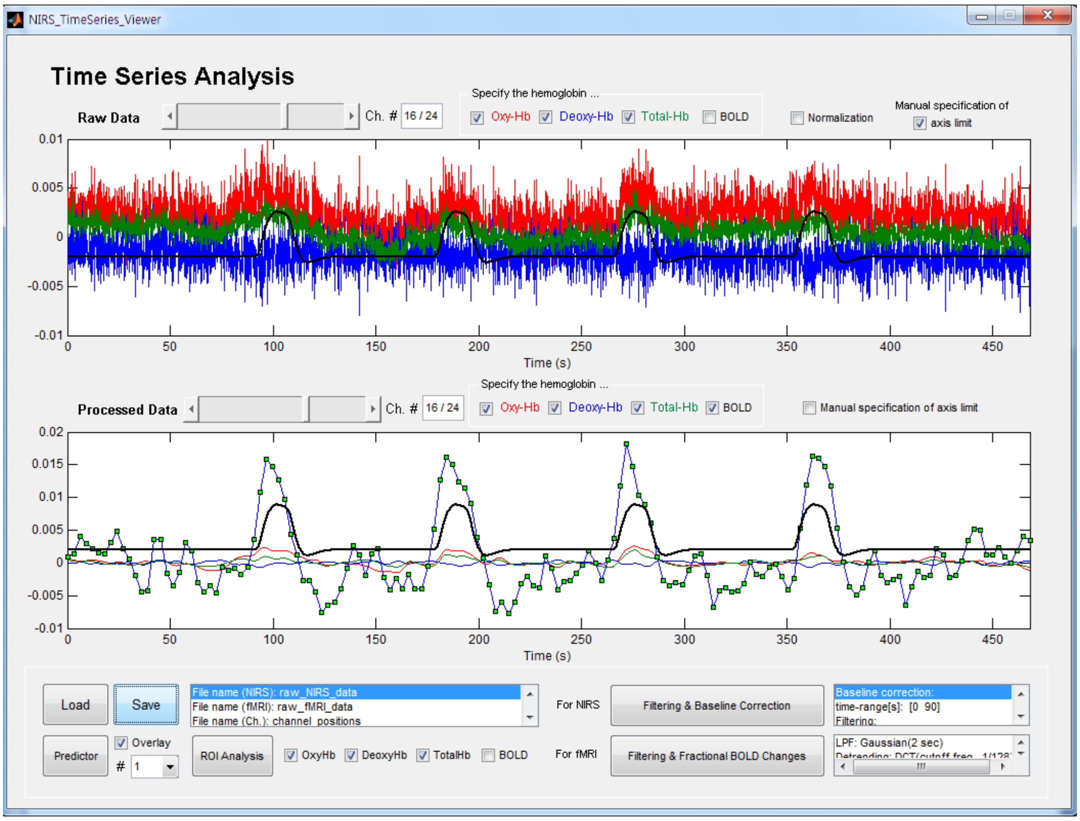

Fig. 14. The window for 'Time Series Analysis' routine. The upper figure is the time series of ΔHbO, ΔHbR, ΔHbT, and BOLD data without filtering. Another figure is the time series of filtered ΔHbO, ΔHbR, ΔHbT (Gaussian smoothing, FWHM 2 sec, wavelet-MDL detrending), and BOLD relative to its baseline (Gaussian smoothing, FWHM 2 sec, DCT-based detrending, cut-off: 128 sec). Red line is ΔHbO, blue line is ΔHbR, green line is ΔHbT, blue line with green marker is BOLD, and black line is a predicted model.

1. Select the 'Time Series Analysis' button in the main panel. The NIRS_TimeSeries_Viewer window will then open.
2. Select 'Load' button and the input window will then open. Highlight 'Loading?' and select the system of measurements whose data will be loaded.

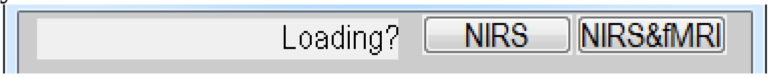

If 'NIRS', use the file selector to choose a file containing the changes in hemoglobin concentrations obtained by the 'Convert' routine,
e.g.,…\Sample_data\CMRO2_Est\raw_NIRS_data.mat

If 'NIRS&fMRI',
1) Use the file selector to choose a file containing the changes in hemoglobin concentrations for NIRS analysis and files containing the blood oxygenation level dependent (BOLD) signals for fMRI analysis.
For reading the fMRI data, NIRS-SPM supports *.img and *.mat file format.
Regarding *.img file format, each *.img file should be the individual volume of functional images.
Regarding *.mat file format, *.mat file should include the structure 'fMRI_data' with the field 'bold' (BOLD time-series, array dimension: # of samples x # of channels).
Refer to the …\Sample_data\CMRO2_Est\raw_fMRI_data.mat file.
2) Specify the interscan interval [secs].

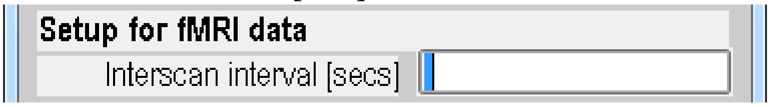

3) Use the file selector to choose a file containing the position of NIRS channels which was obtained by the 'Spatial registration' routine,
e.g. …\Sample_data\CMRO2_Est\channel_positions.mat.

As a result, the time-series of ΔHbO (red line), ΔHbR (blue line), ΔHbT (green line), and BOLD (blue line with green markers) will be plotted on the upper figure.

3.

■ Filtering & Baseline Correction for NIRS data

1) Select the 'Filtering & Baseline Correction' button and the input window will then open
2) Highlight 'Choose the option' and select the processing procedure:
'F': filtering will only be applied,
'B': baseline correction will only be applied,
'B-F': baseline correction and then filtering will be applied,
'F-B': filtering and then baseline correction will be applied.

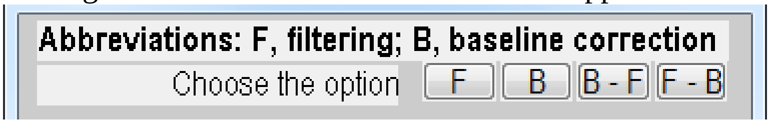

3) If 'B' or 'B-F' or 'F-B' is chosen, the specification of baseline correction starts. Highlight 'Specify the baseline' and choose the answer, e.g., time-range.

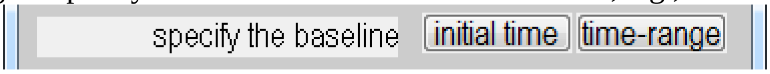

If the ‘initial-time’ is selected, the processed signal y[n] is given by
y[n] = x[n] – x[0], where x(t) is the raw signal.

If the ‘time-range’ is selected, enter the start time of baseline [secs] and the end time of baseline [secs].

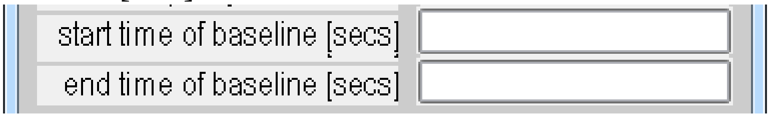

Here, the processed signal y[n] is given by
y[n] = x[n] – mean([x[n_1] x[n_2] … x[n_k]]), where x[n] is the raw signal, index n_1 and n_k denote the start index and end index for averaging the baseline of x[n], respectively.

4) If ‘F’ or ‘B-F’ or ‘F-B’ is chosen, the specification of filtering starts.

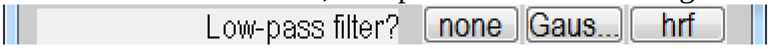

If ‘Gaussian’, specify the filter width (default = 1.5 sec):

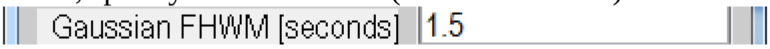

Highlight ‘Detrending?’ and select detrending method.

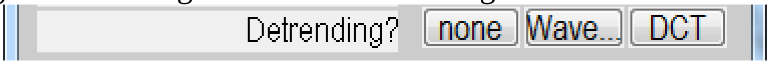

NIRS-SPM provides two options to remove an unknown global trend due to breathing, cardiac, vaso-motion, or other experimental errors.

1- Wavelet-MDL detrending algorithm (Jang et al., 2009)
Wavelet transform is applied to decompose NIRS measurements into global trends, hemodynamic signals, and uncorrelated noise components as distinct scales. The minimum description length (MDL) principle thereupon plays an important role in preventing over- or under- fitting and facilitates optimal model order seelction for the global trend estimate.

In order to perform the wavelet-MDL detrending method, the predicted hemodynamic models (design matrix) should be specified first. Therefore, if you didn’t generate the predictor model, the help dialog will appear and model specification process then start. The details about the model specification are described in the section of ‘Generating the predicted model’ (page 28).

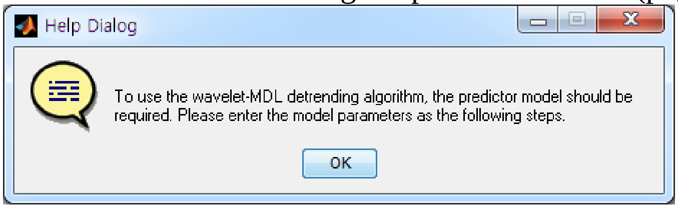

2- DCT-based detrending algorithm (Friston et al, 2006)
The high-pass filter based on a DCT is currently implemented in SPM5 and used as the conventional detrending method.
If DCT is selected, specify the high-pass filter cutoff [seconds] (default = 128).

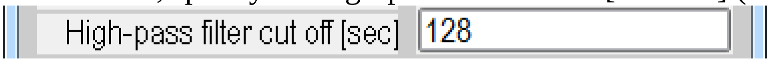

5) After filtering or baseline correction is performed, the results will be displayed on the lower figure.

The detrending result based on Wavelet-MDL vs. the conventional methods.

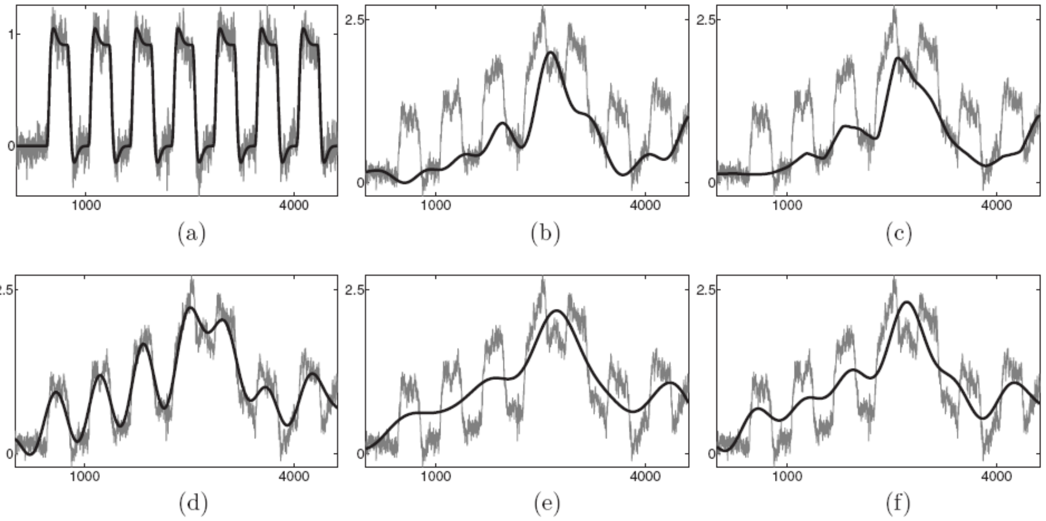

Fig. 15. (a) A synthetic hemodynamic response (black line) and a noise added signal (gray line). (b) The overall simulated signal (gray line) and a ground-truth trend. Trend estimates using (c) Wavelet-MDL, (d) FIR with cutoff frequency 0.02 Hz, (e) FIR with cutoff freuquency 0.015 Hz, and (f) DCT with cutoff frequeucy 0.015 Hz. Wavelet-MDL gives a closer estimate for the unknown trend (Jang et al., 2009)

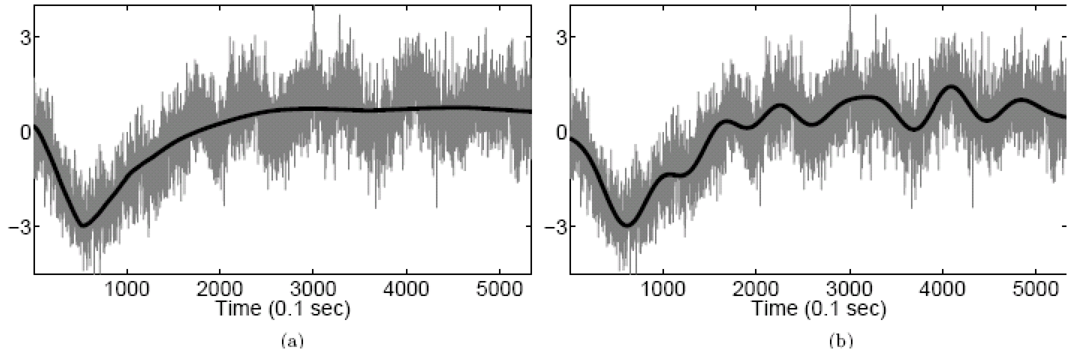

Fig. 16. Oxy-hemoglobin measurement and estimated trend with (a) Wavelet-MDL, and (b) high-pass filter based on a DCT. The task related signals are not removed for the case of Wavelet-MDL. (Jang et al, 2009)

Please refer to our paper (Jang et al., 2009) for details.

- Filtering & Baseline Correction for fMRI data

  Procedures for filtering and baseline correction of fMRI data are same as those for NIRS data.

  Note: As a result of baseline correction, fractional BOLD changes are obtained as follows:

$$\frac{\Delta BOLD[n]}{BOLD_0} = \frac{BOLD[n] - mean([BOLD[n_1], BOLD[n_2] \ldots BOLD[n_k]])}{mean([BOLD[n_1], BOLD[n_2] \ldots BOLD[n_k]])}$$

where BOLD[n] is the BOLD time-series, index n_1, and n_k denote the start index and end index for averaging the baseline of BOLD[n], respectively.

■ Generating the predicted model

1) Select the 'Generate the predicted model' and the input window will then open.
2) Highlight 'specify design in' and choose the units of design, e.g. secs.

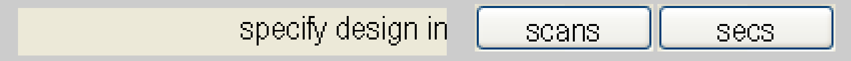

The onsets and durations of events or blocks can be specified in either scans or seconds.

3) Highlight '(Multiple) Conditions – names, onsets, durations. load *.mat file?' and choose yes/no, e.g. no.

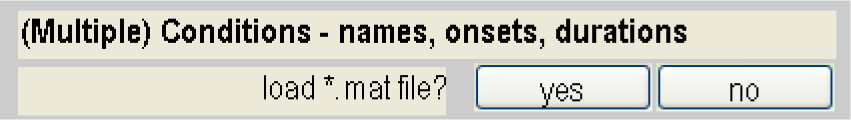

Simultaneous entry of multiple condition names, onsets, and durations using *.mat file is allowed. This option can be used to load all the required information (e.g. condition names, onset, and durations) in one-go. You will first need to create *.mat file containing the relevant information. This *.mat file must include the following cell arrays (each 1 x n) : name, onsets, and durations. Please refer to the sample file; e.g.,…\Sample_data\NIRS_data_file\sample_multiple_condition.mat.

4) Highlight 'number of conditions/trials' and enter the number of conditions, e.g., 1. Any number of condition (event or block) types can be specified. Block and event-related responses are modeled in exactly the same way by specifying their onsets [in terms of onset times] and their durations.
5) Highlight 'name for condition/trial 1?' and enter the condition name, e.g., right finger tapping.
6) Highlight 'vector of onsets – right finger tapping' and enter a vector of onset times for the experiment conditions, e.g., 90 177 264 351.
7) Highlight 'duration[s] (events = 0)' and enter the stimulus durations, e.g., 15 * ones(4,1).

An example of block design sequence consists of 15 s of task (T) and 72 s of rest (R_b) in one cycle. The full experimental run consists of 90 s of rest (R_s), followed by four task and rest cycles, followed by an additional 30 s of rest(R_e); R_s-((T-R_b) x 4 repeat)-R_e. The total recording time is 468 s.

Block and event-related responses are modeled by the exactly same way but specified by their different durations.

(a) If the duration is less than the interscan interval and the unit for design is scans, the duration should be specified with 0.

(b) If the duration is less than 1 second and the unit for design is secs, the duration should be specified with 0.

(c) If you enter a single number for the durations, it will assume that all trials conform to this duration.

(d) If you have multiple different durations, then the number must match the number of onset times.

8) The predicted model is then overlaid with NIRS time series.

Note that in the NIRS data from Hitachi ETG-4000 and ISS Imagent™ system, there is marker column that contains the vector of the onsets and durations. So, NIRS-SPM provides the function to automatically read the vector of onsets and durations from that data and generate the predicted model response.
Highlight ‘specify the onset vector?’ and choose the automatically/manually.

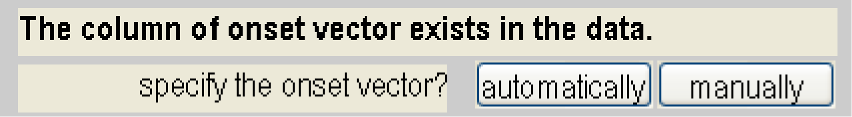

If ‘automatically’ is selected, the vector of onsets and durations will be automatically read and the predicted model response will be then generated.
If ‘manually’ is selected, the specification process of aforementioned parameters will start.

- ROI analysis plotting the average time series among the certain channels or certain time-range.

1) Select the hemoglobin species of interest using checkbox.
2) Select the ‘ROI Analysis’ button and the input window will then open.
3) Highlight ‘specify the data’ and choose the data for ROI analysis, e.g., processed.

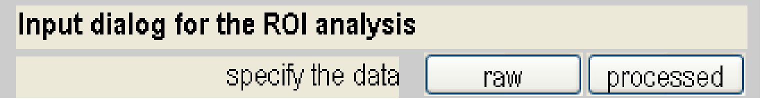

The ‘processed data’ means the filtered data or baseline corrected data.
4) Highlight ‘specify channels of interest’ and enter the channels of interest e.g., 16 19 22.

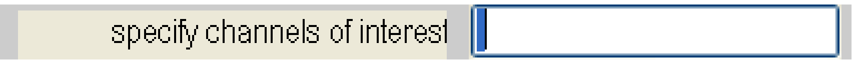

5) Highlight ‘specify design in’ and choose the unit of design, e.g., secs.
6) Highlight ‘vector of onsets’ and enter a vector of onset times within interest, e.g., 102 189 276 363
7) Highlight ‘average duration’ and enter the duration for averaging time-series, e.g., 87

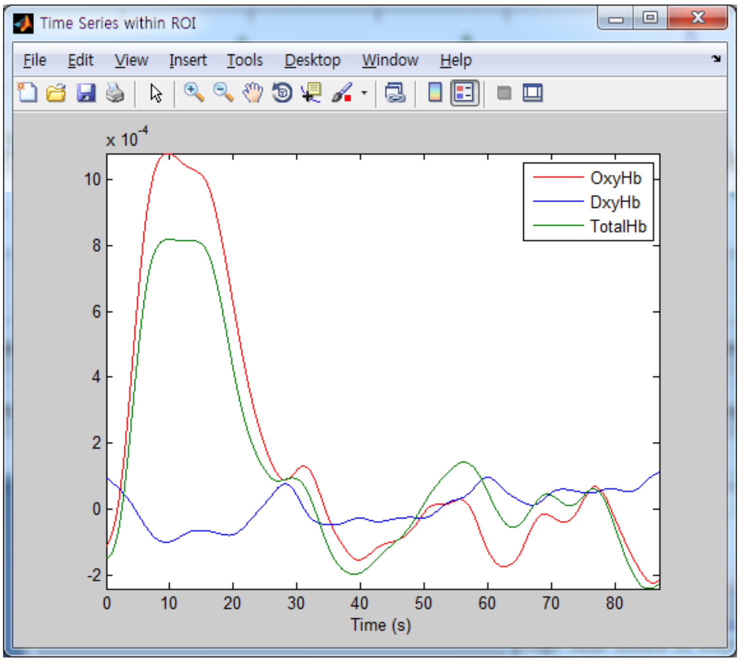

Fig.17. ROI analysis result; average time series of oxy-, deoxy, and total-hemoglobin within certain channel and certain time-range

4. After finishing the process, select the ‘Save’ button and save the processed NIRS and fMRI data as ‘*.mat’ files, e.g. …\Sample_data\CMRO2_Est\filtered_NIRS.mat, filtered_fMRI.mat.
   Note:
   In plotting the raw data, there is an option to normalize the time-series. When you select the ‘Normalization’ checkbox, data which have been normalized to its maximum intensity will be plotted on the upper figure.
   For better visual comparison, axis scaling is available. When you select the ‘Manual specification of axis limit’, the input dialog for specification of x-axis and y-axis limit will open. Enter the x-axis limit or y-axis limit you prefer to change. If you want to change one axis limit, leave the input of another axis limit blank.

■ $CMRO_2$ estimation without hypercapnia

Estimation of the cerebral metabolic rate of oxygen ($CMRO_2$) and cerebral blood flow (CBF) is important to investigate the neurovascular coupling and physiological components in blood oxygen level dependent (BOLD) signals quantitatively. Conventional methods for estimating $CMRO_2$ requires a hypercapnia condition and have many assumed model parameters. In this routine, a novel method to estimate $CMRO_2$ without hypercapnia is implemented using simultaneous measurements of NIRS and fMRI. Using the optimization framework, many assumed parameters such as baseline hemoglobin concentrations and hypercapnia can be readily estimated, which promise more accurate estimation of $CMRO_2$ and CBF (See Tak et al., 2010).

1. Select the 'CMRO2/CBF Estimation' button in the main panel. The 'CMRO2_Est' window will then open.
2. In the 'CMRO2_Est' window, select the 'Load NIRS & fMRI data/Setup model parameters' button and use the file selector to choose a file which contains the NIRS data filtered/baseline corrected using the 'Time Series Analysis' routine, e.g., …\Sample_data\CMRO2_Est\filtered_NIRS.mat.
   Then, the information of parameters for filtering and baseline correction will be displayed in the input window automatically.
   <u>Note: The unit of hemoglobin concentration changes which will be used for further analysis in this routine should be micromol [uM].</u>
3. Use the file selector to choose a file which contains the fMRI data filtered/baseline corrected using the 'Time Series Analysis' routine,
   e.g.,…\Sample_data\CMRO2_est\filtered_fMRI_data.mat
   Then, the information of parameters for filtering and baseline correction will be displayed in the input window automatically:
   Note: The quantities of fMRI data should be the fractional BOLD changes, $\Delta BOLD/BOLD_0$.

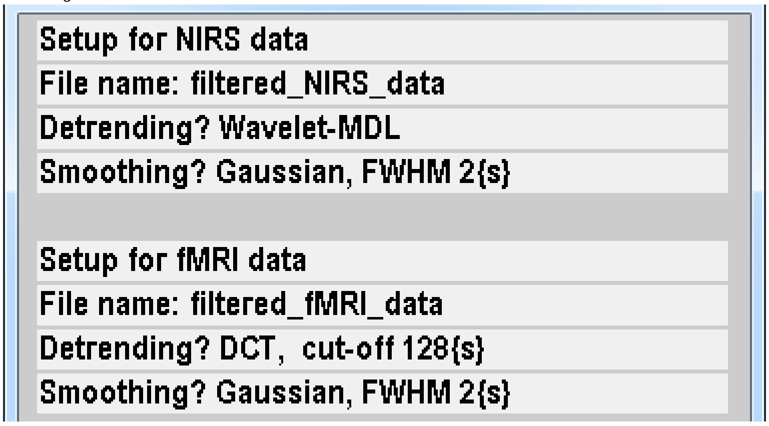

4. Highlight the 'Channels within ROI:' and enter the channels within the region of interest (ROI), e.g. 16 19 22 (This ROI was selected as the HbT activation area ($p< 0.01$, tube formula correction) which overlapped with the primary motor cortex). To help find the channel positions on the brain, the 'NIRS_RegistrationResult_Viewer' will open automatically. CBF and $CMRO_2$ time-series will be estimated on the

corresponding channels within ROI.

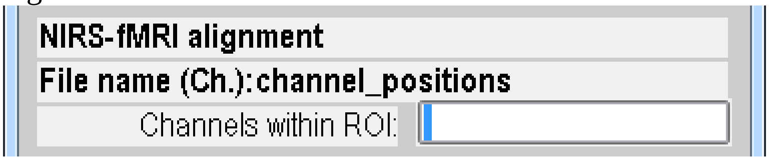

5. Highlight ‘vector of onsets [secs]’ and enter a vector of stimulus onset times for the experimental conditions, e.g., 90 177 264 351.
6. Highlight ‘stimulus duration [secs]’ and enter the stimulus durations, e.g., 15 * ones(4,1).
   An example of block design sequence consists of 15 s of task (T) and 72 s of rest (R_b) in one cycle. The full experimental run consists of 90 s of rest (R_s), followed by four task and rest cycles, followed by an additional 30 s of rest (R_e): R_s-((T-R_b) x 4 repeat)-R_e. The total recording time is 468 s.

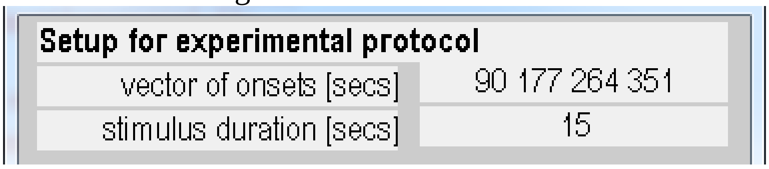

   This setup for experimental protocol is required for a wavelet-based adaptive averaging method to estimate a hemodynamic response function (HRF) (See the appendix of Tak et al., 2010 for details).
   <u>Note that the current version of wavelet-based adaptive averaging method is available on application of the data of an experiment with only one condition.</u>
7. Highlight ‘Search range from model parameters – Hypercapnia (%)’ and enter the search range for hypercapnia calibration parameter, e.g. 0 10.
8. Highlight ‘Beta’ and enter the search range for β parameter, e.g. 1 2.
   Note: β parameter is used in the Davis et al.(1998)’s model as follows:

$$\frac{\Delta\mathrm{BOLD}(t)}{\mathrm{BOLD}_0} \cong -\mathrm{TE}\Delta R^*_{2,\mathrm{HbR}}(t)$$

$$\cong H\left(1-\mathrm{rCBV}(t)\left(\frac{[\mathrm{HbR}(t)]_v}{[\mathrm{HbR}]_{v0}}\right)^{\beta}\right)$$

9. Highlight ‘CBV_0’ and enter the search range for total baseline blood volume, CBV_0, e.g. 40 140.
10. Highlight ‘SO_2 (%)’ and enter the search range for baseline oxygen saturation, SO_2, e.g. 55 80.
11. Highlight ‘Venous deoxy-Hb ratio’ and enter the search range for venous deoxy-hemoglobin ratio, e.g. 0.5 1.5.
12. Highlight ‘Venous total-Hb ratio’ and enter the search range for venous total-hemoglobin ratio, e.g., 0.5 1.5.
13. Highlight ‘Partial volume factor’ and enter the partial volume factor, e.g., 6.2.
    The optical and MR measurements reflect the changes over the different volume of tissue, which lead to different partial volume effect. Since the partial volume effect affects the accuracy of parameter estimation, it should be corrected. By default, NIRS-SPM employs the partial volume correction factor p of 6.2 from Durduran et al. 2004.

Note: Depending on the search range (especially partial volume factor), $CMRO_2$/CBF time-series which obtained from BOLD biophysical model can be reversed.

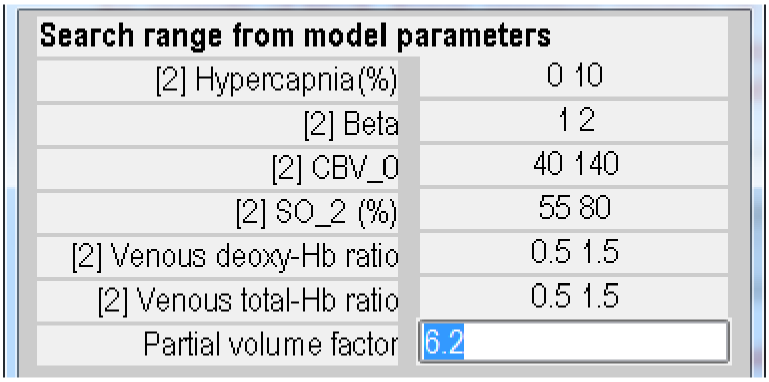

14. Highlight ‘CBF-CBV relationship, Alpha (CBF=CBV^alpha)’ and enter the power of Grubb et al.(1974) relationship, α, e.g., 0.38.

    Note: the Grubb’s relationship is as follows: $rCBV(t) = rCBF(t)^{\alpha}$

    After a while, results including hemodynamic responses, relative $CMRO_2$/CBF coupling ratio from a NIRS biophysical model (red line) and a BOLD biophysical model (blue line), and estimated CBF (red line) and $CMRO_2$ (blue line) will be obtained as shown in Fig. 18.

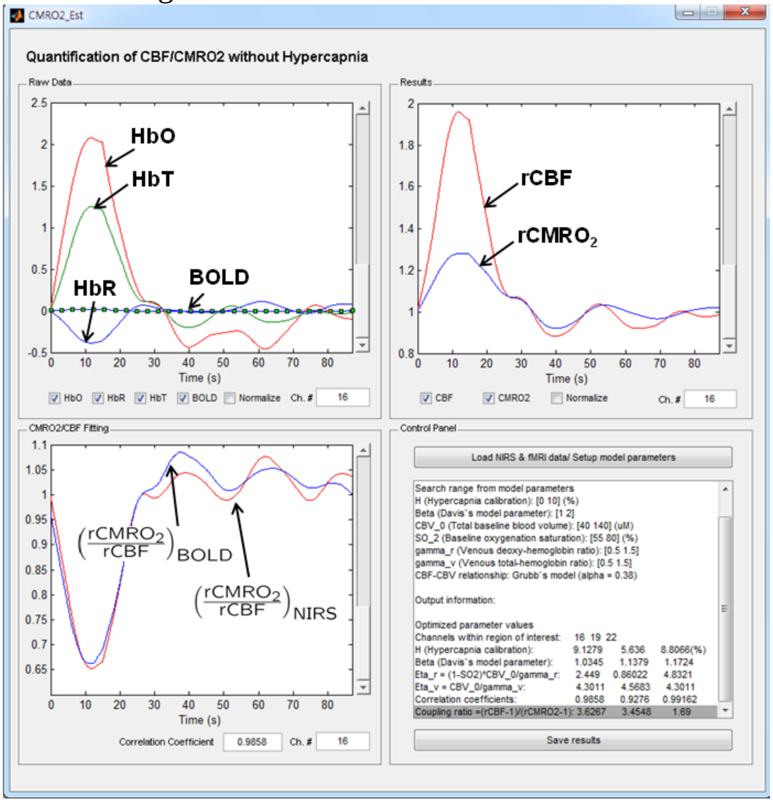

Fig.18. The window for $CMRO_2$ estimation without hypercapnia from simultaneous NIRS and fMRI measurements.

15. Select ‘Save results’ button and save the processed NIRS and fMRI data as *.mat file, e.g., …\Sample_data\CMRO2_Est\results_rCMRO2_rCBF.mat.

■ *Batch Files (NIRS-SPM v.4)*

We provide batch files for 'Convert', 'Specify 1st level', 'Estimate', and 'Results NIRS steps.

- **'…\batch_file\data_conversion_batch.m'**
  batch script for 'Convert' routine, which reads the optical density or hemoglobin concentration changes from the raw data and converts it to NIRS-SPM format.
- **'…\batch_file\specification_batch.m'**
  batch script for 'Specify 1st level' routine, which specifies the general linear model (GLM) such as the design matrix, temporal filtering, and temporal correlation estimation.
- **'…\batch_file\estimation_batch.m'**
  batch script for 'Estimation' routine, which estimates the GLM parameters and temporal correlation.
- **'…\batch_file\activation_map_batch.m'**
  batch script for 'Results NIRS' routine, which calculates the activation map over the threshold.
- **'…\batch_file\run_all_process_batch.m'**
  run file for all processes including data conversion, model specification, estimation. and activation map.

Detailed usage for each batch script is written in the corresponding source code.

Appendix I - Channel Configuration

Artinis's OXYMON MKIII

4x4

Red circle : illuminator (Tx),
Blue circle : detector (Rx)
Square : channel

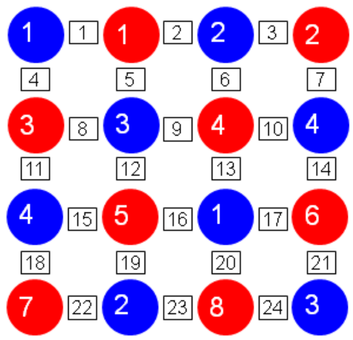

The order of optode positions recorded by 3D digitizer:

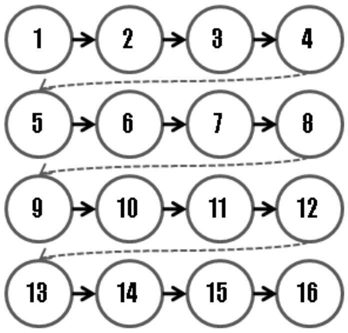

Hitachi's ETG4000

3x11

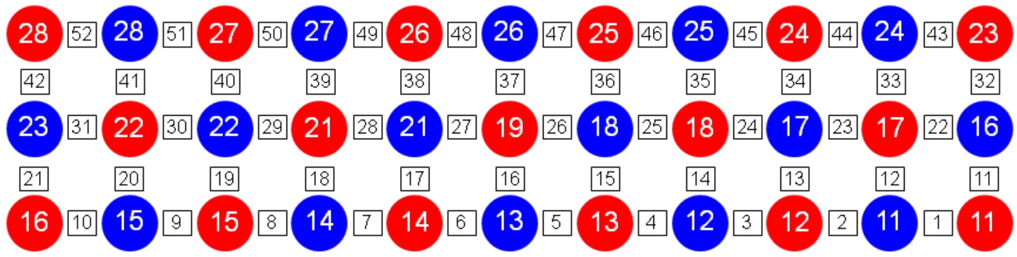

Red circle : illuminator (Tx), Blue circle : detector (Rx), Square : channel
The order of optode positions recorded by 3D digitizer:

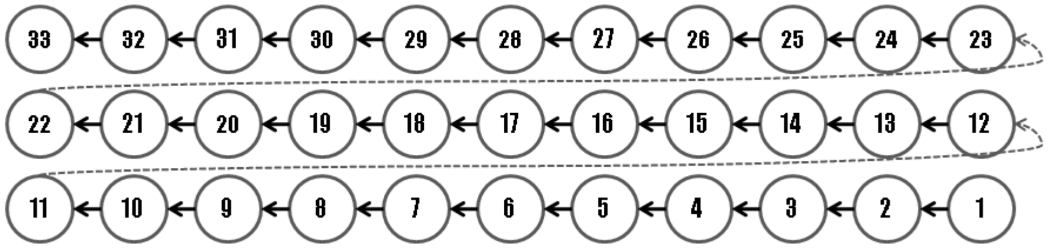

Hitachi’s ETG4000
4x4

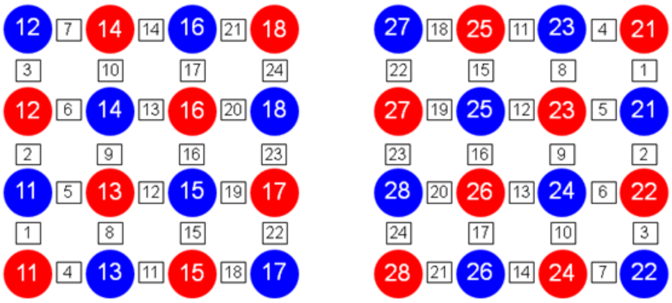

Red circle : illuminator (Tx), Blue circle : detector (Rx), Square : channel
The order of optode positions recorded by 3D digitizer:

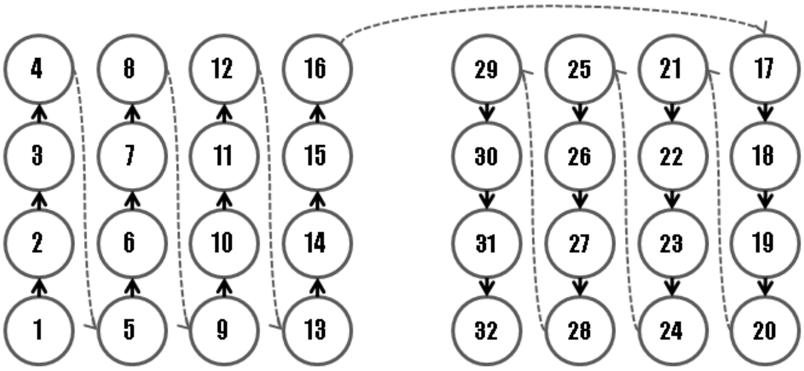

Hitachi’s ETG4000
3x5

Red circle : illuminator (Tx), Blue circle : detector (Rx) Square: channel

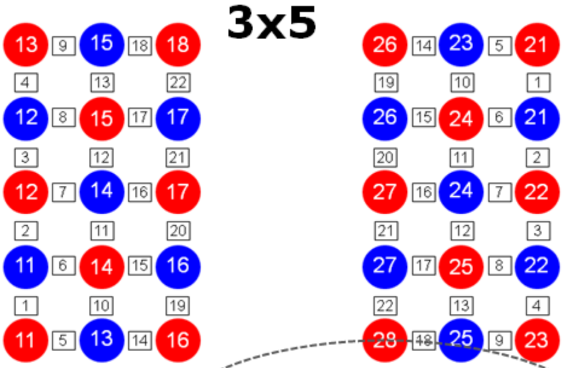

The order of optode positions recorded by 3D digitizer:

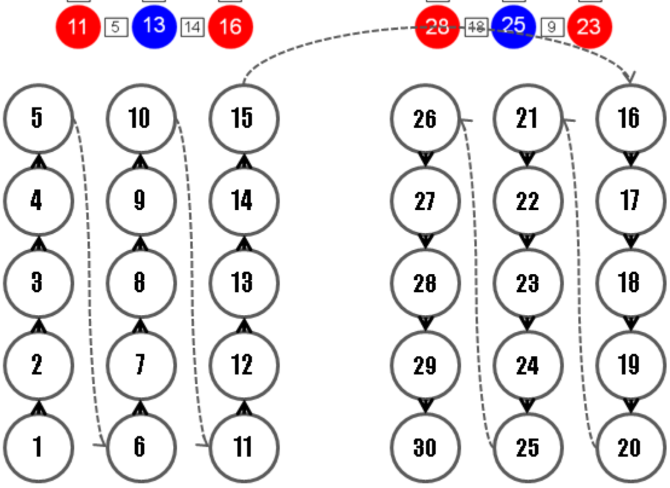

Shimadzu's FOIRE3000
4x4x2

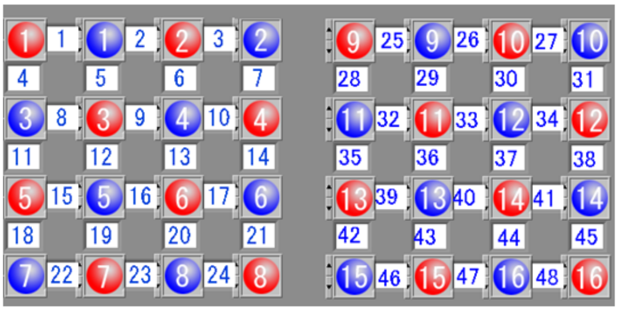

Red circle : illuminator (Tx), Blue circle : detector (Rx)
The order of optode positions recorded by 3D digitizer:

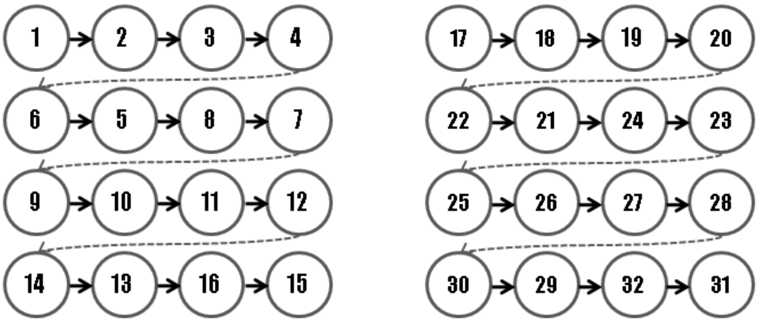

BIOPAC fNIR

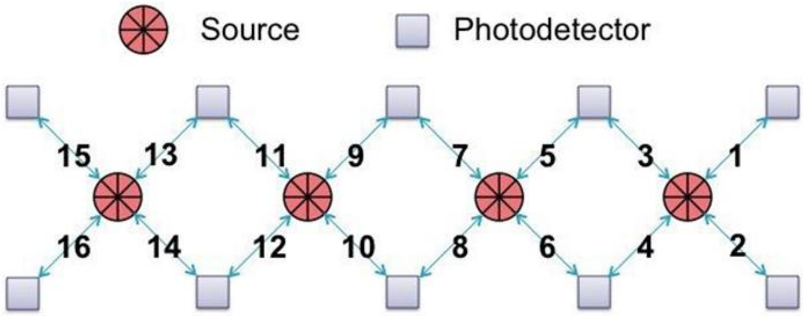

The order of optode positions recorded by 3D digitizer:

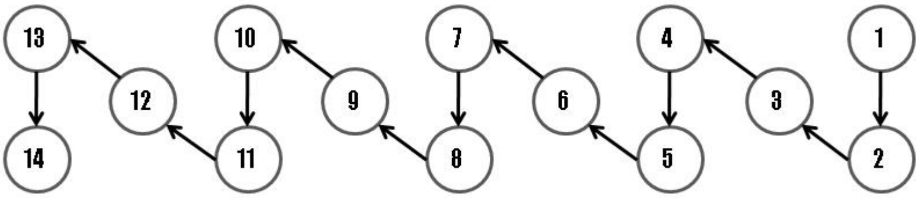

Bibliography

Ye, J.C., Tak, S., Jang, K. E., Jung, J., Jang, J., 2009, NIRS-SPM: Statistical parametric mapping for near-infrared spectroscopy. NeuroImage 44, 428-447.

Jang, K. E., Tak, S., Jung, J., Jang, J., Jeong, Y., and Ye, J.C., 2009, Wavelet-MDL detrending for near-infrared spectroscopy (NIRS). Journal of Biomedical Optics, vol. 14, no. 3, pp. 1-13, May/June 2009.

Tak, S., Jang, J., Lee, K., and Ye, J.C., 2010, Quantification of $CMRO_2$ without hypercapnia using simultaneous near-infrared spectroscopy and fMRI measurements. Phys. Med. Biol 55, 3249-3269.

Tak, S., Yoon, S. J., Jang, J. Yoo, K., Jeong, Y., and Ye, J.C., 2011, Quantitative analysis of hemodynamic and metabolic changes in subcortical vascular dementia using simultaneous near-infrared spectroscopy and fMRI measurements. NeuroImage 55, 176-184.

Li, H., Tak, S., and Ye, J.C., 2012, Lipschitz-Killing curvature based expected Euler characteristics for p-value correction in fNIRS. J. Neurosci. Meth. 204, 61-67.

Ye, J. C., Moulin, P., Bresler, Y., October 2006. Asymptotic global confidence regions for 3-D parametric shape estimation in inverse problems. IEEE Trans. on Image Processing 15 (10), 2904–2919.

Bullmore, E., Brammer, M., Williams, S.C., Rabe-Hesketh, S., Janot, N., David, A., Mellers, J., Howard, R., Sham, P., 1996. Statistical methods of estimation and inference for functional MR image analysis Magn Reson Med 35, 261-277.

Cope, M., Delpy, D. T., May 1988. System for long-term measurement of cerebral blood and tissue oxygenation on newborn infants by near infra-red transillumination. Med. Biol. Eng. Comput. 26, 289–294.

Friston, K.J., Josephs, O., Zarahn, E., Holmes, A.P., Rouquette, S., Poline, J.B., August 2000. To smooth or not to smooth? Bias and efficiency in fMRI time-series analysis. NeuroImage 12, 196-208.

Friston, K.J., Ashburner, J., Kiebel, S., Nichols, T., Penny, W. (Eds.), 2006. Statistical Parametric Mapping: The Analysis of Functional Brain Images. Academic Press, Sandiego, CA, USA.

Horn, B. K. P., April 1987. Closed-form solution of absolute orientation using unit quaternions. J. Opt. Soc. Amer. A 4, 629–642.

Huppert T.J., Diamond, S.G., Franceschini, M.A., Boas, D.A., April 2009. HomER: a review of time-series analysis methods for near-infrared spectroscopy of the brain. Appl. Opt. 48(10), D280-D298.

Koh, P. H., Glaser, D. E., Flandin, G., Kiebel, S., Butterworth, B., Maki, A., Delpy, D. T., Elwell, C. E., November 2007. Functional optical signal analysis: a software tool for near-infrared spectroscopy data processing incorporating statistical parametric mapping. J. Biomed. Opt. 12, 1–13.

Lancaster J.L., Woldorff MG, Parsons LM, Liotti M, Freitas CS, Rainey L, Kochunov PV, Nickerson D, Mikiten SA, Fox PT. Automated Talairach atlas labels for functional brain mapping. Human Brain Mapping 2000; 10: 120-131.

Okamoto, M., Dan, H., Sakamoto, K., Takeo, K., Shimizu, K., Kohno, S., Oda, I., Isobe, S., Suzuki, T., Kohyama, K., Dan, I. 2004, Three-dimensional probabilistic anatomical cranio-cerebral correlation via the international 10-20 system oriented for transcranial functional brain mapping. NeuroImage, 21(1), 99-111.

Plichta, M., Herrmann, M., Baehne, C., Ehlis, A., Richter, M., Pauli, P., Fallgatter, A., 2006. Event-related functional near-infrared spectroscopy (fNIRS): Are the measurements reliable? Neuroimage 31 (1), 116–124.

Plichta, M. M., Heinzel, S., Ehlis, A. C., Pauli, P., Fallgatter, A. J., April 2007. Model-based analysis of rapid event-related functional near-infrared spectroscopy (NIRS) data: A parametric validation study. NeuroImage 35, 625–634.

Rorden, C., Brett, M., 2000. Stereotaxic display of brain lesions. Behav. Neurol. 12, 191–200.

Singh, A.K., Okamoto, M., Dan, H., Jurcak, V., Dan, I., 2005, Spatial registration of multichannel multi-subject fNIRS data to MNI space without MRI. NeuroImage 27(4), 842-851.

Sun, J., Loader, C., 1994. Simultaneous confidence bands for linear regression and smoothing. Ann. Statist 22 (3), 1328–1345.

Shattuck DW, Mirza M, Adisetiyo V, Hojatkashani C, Salamon G, Narr KL, Poldrack RA, Bilder RM, Toga AW. Construction of a 3D probabilistic atlas of human cortical structures. NeuroImage 2007; 39: 1064-1080.

Takemura, A., Kuriki, S., 2002. On the equivalence of the tube and Euler characteristic methods for the distribution of the maximum of Gaussian fields over piecewise smooth domains. Ann. Appl. Probab 12 (2), 768–796.

Tzourio-Mazoyer, N., Landeau, B., Papathanassiou, D., Crivello, F., Etard, O., Delcroix, N., Mazoyer, B., Joliot, M., 2002. Automated anatomical labeling of activations in SPM using a macroscopic anatomical parcellation of the MNI MRI single-subject brain. NeuroImage 15 (1), 273– 289.

Worsley, K. J., Friston, K. J., 1995. Analysis of fMRI time-series revisited again NeuroImage 2, 173–181
